# Roles of microglia in adult hippocampal neurogenesis in depression and their therapeutics

**DOI:** 10.3389/fimmu.2023.1193053

**Published:** 2023-10-10

**Authors:** Shaoyi Fang, Zhibin Wu, Yali Guo, Wenjun Zhu, Chunmiao Wan, Naijun Yuan, Jianbei Chen, Wenzhi Hao, Xiaowei Mo, Xiaofang Guo, Lili Fan, Xiaojuan Li, Jiaxu Chen

**Affiliations:** ^1^ Formula-Pattern of Traditional Chinese Medicine, School of Traditional Chinese Medicine, Jinan University, Guangzhou, China; ^2^ Shenzhen People’s Hospital, 2^nd^Clinical Medical College, Jinan University, Shenzhen, China; ^3^ School of Traditional Chinese Medicine, Beijing University of Chinese Medicine, Beijing, China

**Keywords:** depression, microglia, adult hippocampal neurogenesis, antidepressant, neurogenesis

## Abstract

Adult hippocampal neurogenesis generates functional neurons from neural progenitor cells in the hippocampal dentate gyrus (DG) to complement and repair neurons and neural circuits, thus benefiting the treatment of depression. Increasing evidence has shown that aberrant microglial activity can disrupt the appropriate formation and development of functional properties of neurogenesis, which will play a crucial role in the occurrence and development of depression. However, the mechanisms of the crosstalk between microglia and adult hippocampal neurogenesis in depression are not yet fully understood. Therefore, in this review, we first introduce recent discoveries regarding the roles of microglia and adult hippocampal neurogenesis in the etiology of depression. Then, we systematically discuss the possible mechanisms of how microglia regulate adult hippocampal neurogenesis in depression according to recent studies, which involve toll-like receptors, microglial polarization, fractalkine-C-X3-C motif chemokine receptor 1, hypothalamic-pituitary-adrenal axis, cytokines, brain-derived neurotrophic factor, and the microbiota-gut-brain axis, etc. In addition, we summarize the promising drugs that could improve the adult hippocampal neurogenesis by regulating the microglia. These findings will help us understand the complicated pathological mechanisms of depression and shed light on the development of new treatment strategies for this disease.

## 1 Introduction

Depression is one of the most common psychiatric disorders affecting humans. Its clinical symptoms include impaired sociability, anhedonia, behavioral despair, loss of appetite, sleep problems, suicidal tendencies, and anxiety, which lead to a severe decline in quality of life (1). Depression is considered a common mental disorder with a complex and multifactorial etiology. At present, the prognosis of clinical antidepressant treatment is poor, and drug development for depression has shown high failure rates in clinical trials (2). Therefore, the underlying mechanisms of depression remain to be further explored.

The pathogenesis mechanisms associated with depression have been reported including monoamine neurotransmission, dysregulation of the hypothalamic-pituitary-adrenal axis (HPA axis), synaptic remodeling, inflammation, neurogenesis, etc (3), among which neurogenesis is one of the most important aspects. Neurogenesis is the process by which value-added neural precursor cells differentiate to produce new mature neurons and integrate into an established neural network to function (4). There are two major reservoirs of neurogenesis: the subventricular zone (SVZ) of the lateral ventricle and the dentate gyrus (DG) of the hippocampus (5). Accumulating evidence has shown that impaired hippocampal neurogenesis is a critical factor in the pathogenesis of depression in rodents (6–8). In addition, promoting effective neurogenesis is emerging as an important strategy for the treatment of depression, as dead and lost neurons in the lesion require replacement by newborn neurons to replenish and reestablish neuronal connections. For instance, first-line antidepressants on the market, such as fluoxetine and paroxetine, can promote the value-added and survival of hippocampal precursor cells in rodents, which in turn can have antidepressant effects (9). Although significantly reduced hippocampal volume has been found in the brains of depressed patients using MRI (10), evidence for impaired hippocampal neurogenesis in depression patients is lacking. Particularly, single-cell technologies, mainly single-cell RNA sequencing, are novel technologies to analyze the genome sequence in a single cell, which enables comprehensive and high-resolution cell type determination and identifying new cell markers, offering new possibilities to address biological and medical questions (11). In a recent study, hippocampal immature neurons in human present across the entire human lifespan have been found using the new single-cell technologies (12), suggesting that the damage to adult hippocampal neurogenesis in depression patients will one day be visible with technological innovations in the neurological field. The present study suggests that the mechanism of impaired adult neurogenesis in depression is related to the abnormal activation of microglia.

Evidence indicates that immunoreactions can directly or indirectly affect the neurobiological processes of mental disorders (13–15). Accumulating evidence indicates that neuroinflammation plays a leading role in depression. For example, it has been demonstrated that there is a strong association between the levels of inflammatory factors in the peripheral blood and cerebrospinal fluid (CSF) in depression patients (16). Studies in rodents have also shown that immune challenges can induce depressive-like behaviors (17, 18). Microglia serve as immune cells in the central nervous system (CNS) and play dominant roles in monitoring the microenvironment for any stimuli or injury that may be harmful. When pro-inflammatory cytokines are consistently increased in the circulatory system of patients with depression, microglia are quickly activated and subsequently secrete a variety of non-discriminative harmful factors, finally leading to neuronal damage and the pathogenesis of depression. Importantly, neuroinflammation, mainly triggered by microglia, contributes to reduced hippocampal neurogenesis, which is important for the development of depression. However, the mechanism of the reduction in neuroinflammation-induced hippocampal neurogenesis in depression remains to be further elucidated, according to recent discoveries.

Therefore, in order to review the roles and mechanisms of microglia in the neurogenesis of depression, we first introduce neurogenesis, microglia, and the role of both in depression. Then, we systematically discussed the possible mechanisms of how microglia modulate the adult hippocampal neurogenesis in depression and summarized the promising drugs targeting microglia to alter neurogenesis for treating depression and their potential regulation mechanism, which will help in understanding the pathogenesis of depression and promoting the development of new and exciting treatments for depression.

## 2 Neurogenesis

### 2.1 Introduction of neurogenesis

Neurogenesis is the process by which new neurons be generated by neural stem cells (NSCs), promoting the structural plasticity of the brain (19). Although neurons in the adult brain do not regenerate or replenish in the traditional view, recent decades have seen the publication of numerous studies demonstrating that a large majority of mammalian species retain the capacity for neurogenesis in the CNS into adult life. It is currently understood that the adult mammalian brain has two major reservoirs of neural stem cells (NSCs), called “neurogenic niches” in the SVZ of the lateral wall of the lateral ventricle and the subgranular zone (SGZ) of the DG of the hippocampus (20). Under resting conditions, neurogenesis is restricted in the above two neurogenic niches, while under pathological conditions, neural precursor cells generated in the SGZ and SVZ need to migrate to the olfactory bulb and the subgranular zone of the DG of the hippocampus to compensate for neuronal loss in the brain to maintain hippocampal structural and functional plasticity (21). Although the process of neurogenesis is sophisticated, it can be summarized in four phases: In the first stage, NSCs divide and proliferate to form a pool of NSCs, which can differentiate to produce neuroblasts and further differentiate into immature neurons. In the second stage, neuroblasts and immature neurons located in the subgranular region migrate to the granular cell layer. However, due to the mature neuronal structures not yet being formed, the axons and dendrites of immature neurons are short. In the third stage, in order to establish correct synapses with other neurons, immature neurons differentiate into mature neurons with intact neuronal structures and neurites. Meanwhile, dendrites extend into the molecular layer and axons extend to the CA3 subfield. Finally, the dendrites and axons of mature neurons establish synaptic connections with other neurons, and these synaptic connections integrate with pre-existing circuits to ensure the normal functioning of the CNS (22). Since the hippocampus is an important structure involving humans’ learning, memory, and mood regulation (23, 24), and the DG is one of the core regions for neurogenesis, neurogenesis in the hippocampus has been implicated in the process of cognitive functions and emotion. Indeed, it has been found that adult-born neurons in the DG contribute to learning and memory, including cognitive flexibility, emotional memory, spatial navigation, novelty detection, pattern separation, stress response, and emotion (25). Thus, the regulation of adult neurogenesis has a vital impact on the treatment of some neuropsychiatric diseases.

### 2.2 Adult hippocampal neurogenesis and depression

**Figure 1:**
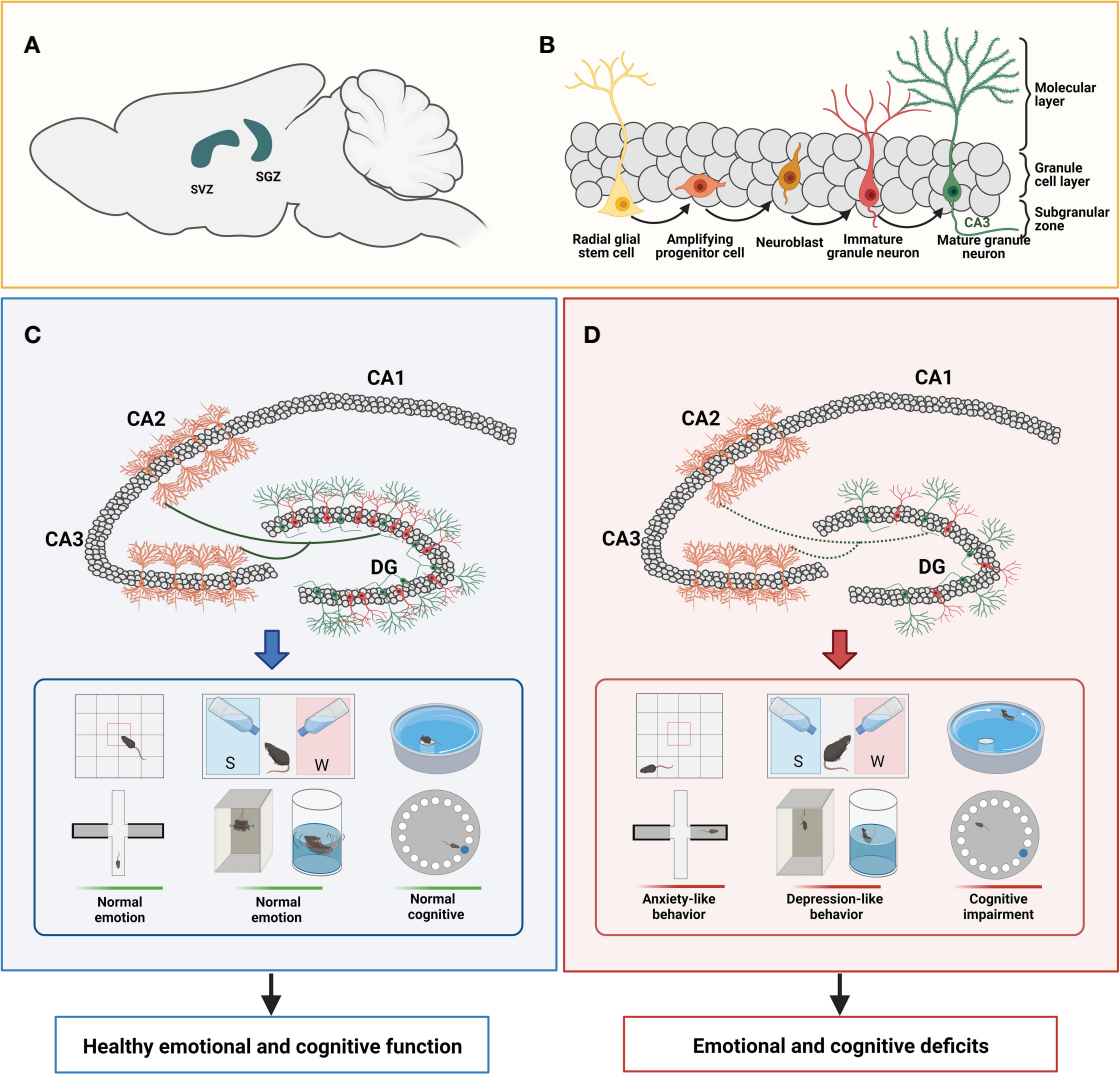
Neurogenesis and its role in learning and memory, cognitive function, and emotion. **(A)** The area of neurogenesis is limited to the SVZ of the lateral ventricle and the SGZ of the hippocampus. **(B)** Radial glial stem cells in the subgranular zone produce neural stem cells (NSCs). Amplifying progenitor cells located in the subgranular zone arise from the asymmetric division of NSCs and differentiate into neuroblasts. Neuroblasts migrate into the granule cell layer to develop into immature granule neurons. Immature neurons grow into mature neurons, and their dendritic trees gradually extend to the molecular layer and axons extend to the CA3 subfields. **(C)** Under normal conditions, the axons of new granule neurons located in the DG area will extend to the CA3 and CA2 subfields to establish synaptic contacts with pyramidal neurons and integrate the pre-existing circuits, playing an important role in behaviors associated with the hippocampus. Rodents with normal neurogenesis have typical responses in some emotional tests (such as OFT, EPM, SPT, TST, and FST) and cognitive tests (such as MWM and Barnes maze). **(D)** In some neuropsychiatric diseases, hippocampal neurogenesis is impaired, manifested by decreased newborn neurons and impaired synaptic connections with pyramidal neurons in the CA3 and CA2 subfields. Compared to normal rodents, rodents with impaired neurogenesis exhibit anxiety-like behaviors (such as reduced entry times and residence times in the central zone in the OFT; reduced the number and frequency of exploring the open arm, and prefer to stay in the closed arm in the EPM) or depression-like behaviors on certain mood tests (such as inactive behaviors in the FST and TST; anhedonia in SPT), and impaired social cognition on some cognitive tests (such as unable to locate the location of the hidden platform in the MWM and the location of the markers in the Barnes maze). SVZ subventricular zone, SGZ subgranular zone, DG dentate gyrus, OFT open field test, EPM Elevated Plus Maze, SPT Sucrose Preference Test, TST Tail Suspension Test, FST Forced Swimming Test, MWM Morris Water Maze.

Abnormalities in different stages of neurogenesis can lead to the development of depression (26). First, in patients with depression, the number and differentiation capacity of NSCs in the hippocampus decrease, which may lead to a decrease in neurons and dysfunction, which affects emotion regulation and cognitive function in patients with depression (27, 28). The antidepressant fluoxetine can increase the proliferative capacity of NSCs and the expression of Bcl-2, thereby preventing apoptosis through the activation of Bcl-2, promoting the development of synapses and the differentiation of serotonin neurons (29). This study reveals a new antidepressant drug treatment mechanism that promotes the development and differentiation of NSCs by stimulating Bcl-2 expression, thereby helping to treat depression. Secondly, when there are problems with the maturity of newborn neurons in the hippocampus, it may negatively affect behavioral functions such as learning, memory, and emotional control (30). Previous studies have shown that antidepressants can promote the increase of new neurons. For instance, many studies have shown that antidepressants including fluoxetine, paroxetine, tranylcypromine, and reboxetine reverse hippocampal volume loss in patients with depression as well as depression in rodents (8, 31, 32). Furthermore, the proliferation rate of newborn neurons, which is promoted by fluoxetine in the hippocampus, is synchronized with the clinically observed delay time of the therapeutic effect (33). It has been shown that newborn cell proliferation in the adult hippocampus decreases when exposed to chronic stress (34). Interestingly, the presence of newborn neurons was sufficient to cause anti-reversal and remission in mouse models of depression (35, 36). In addition, previous studies have shown that in the early neuronal development stage, neurons with deletion of the TLR7 gene show downregulation of gene expression related to neurodevelopment, synaptic organization, and activity, while mice with deletion of the TLR7 gene show significant behavioral changes in anxiety, aggression, smell, and situational fear memory. The electrophysiological analysis further revealed that loss of *TLR7* led to impaired hippocampal long-term enhancement (37). What’s more, recent studies showed that if the newborn granule cells are damaged, it causes damage to neurons connected to CA3 in the DG network, which affects the information coding in the downstream hippocampal region, which may lead to the onset and progression of depression (38, 39). The above implies that impaired neuronal maturity can also lead to depression. Therefore, this current research has indicated that diminished adult hippocampal neurogenesis may be associated with the development of depression, and that it might be one of the ways to reverse or prevent diminished hippocampal neurogenesis in adults with antidepressants (**Figure 1**).

## 3 Microglia

### 3.1 Introduction of microglia

Microglial originate from the embryonic mesoderm, primarily derived from myeloid progenitors within the yolk sac (40). During early embryonic development, these cells persistently proliferate in the brain and are regulated by various molecular factors, including but not limited to transcription factors, growth factors, chemokines, MMPs, and microRNAs (41). The exact numbers or proportions of microglia in the hippocampus can vary, influenced by factors such as age and health (42). Generally, the hippocampus exhibits a relatively high microglial density compared to other brain regions, reflecting its vital role in cognitive function (43). In the hippocampus, microglia constitute a slightly higher proportion, accounting for approximately 10 to 15 percent of the total cell population, whereas in other brain regions, this proportion typically ranges from 5 to 10 percent (43). Microglia are the leading type of immune cells in the CNS and are in charge of modulating inflammation. Originally, microglia were thought to exist in a quiescent or resting state when characterized by small stationary soma, with smaller cell bodies and highly branched protrusions (44). Normally, the highly branched resting state of microglia provides a highly dynamic and efficient monitoring system for the brain (Physiology of Microglia) (45, 46). Conversely, when pathological stimulation occurs, microglia are rapidly activated. There are three main activated microglial morphotypes in current reports: hyper-ramified, phagocytic, and amoeboid types (47–49). When the brain microenvironment changes, the resting microglia react by transitioning into “hyper-ramified” morphotypes, which are often defined by longer, thicker, more abundant processes that attach to larger, lobular, and irregularly shaped cell bodies (50, 51). Once the changes or stimuli intensify, the hyper-ramified microglia shift into phagocytic and even amoeboid types, which show much fewer, albeit thicker and shorter, processes, and much rounder, larger, and more regularly shaped cells with no processes, respectively (52). Alternatively, the activated microglia, similar to peripheral macrophages, have two primary polarization phenotypes: M1 (classical activation) and M2 (alternative activation) (53). M1-phenotype microglia are classically activated microglia that can express surface markers such as cluster of differentiation 86 (CD86) and inducible nitric oxide synthase (iNOS) (54, 55). M1-phenotype microglia mainly play a pro-inflammatory role and promote the synthesis of inflammatory mediators such as tumor necrosis factor-alpha (TNF-α), interleukin-1β (IL-1β), and iNOS, which can promote chronic neuroinflammation, phagocytosis, oxidative stress, and neurodegeneration and inhibit regeneration (56). Therefore, M1 microglia often have obvious neurotoxic effects (57). In contrast, the M2-phenotype microglia are alternatively activated microglia that can express surface markers such as cluster of differentiation 206 (CD206) and cluster of differentiation 163 (CD163), which release neuroprotective and anti-inflammatory cytokines, such as interleukin-10 (IL-10) and transforming growth factor-β (TGF-β), to exert anti-inflammatory effects, promote wound healing, neuroprotection, and tissue repair (58). Therefore, M2 microglia often have neuroprotective effects (59). Furthermore, many studies have reported three main sub-phenotypes (M2a, M2b, and M2c) in M2 microglia, corresponding to different stimulations and functions (42). The M2a phenotype can be activated by interleukin-4 (IL-4) and interleukin-13 (IL-13), resulting in the secretion of IL-10, chemoattractant cytokine ligand 17 (CCL17), and chemoattractant cytokine ligand 18 (CCL18) to clear apoptotic cells, promote cell debris removal, and contribute to neuroprotection (56, 60, 61). The M2b phenotype can be activated by toll-like receptor (TLR) ligands and immune complexes, which secrete TNF-α, interleukin 1 (IL-1), and interleukin 6 (IL-6). These cytokines support immune regulation, inflammation inhibition, B cell conversion and antibody production, and recruitment of regulatory T cells (62, 63). The M2c phenotype can be activated by anti-inflammatory IL-10 and corticosteroids, which in turn release many more anti-inflammatory cytokines, such as IL-4, and increase growth factors, such as TGF-β, to promote nerve regeneration (64, 65). Usually, an imbalance of M1 and M2 polarization occurs in neurodegenerative or neuro-damaged illnesses, such as depression, stroke, and Alzheimer’s disease, and therapeutic candidates that target modulating the activation of M1 and M2 polarization appear promising for these diseases (66).

### 3.2 Microglial activation and depression

**Figure 2:**
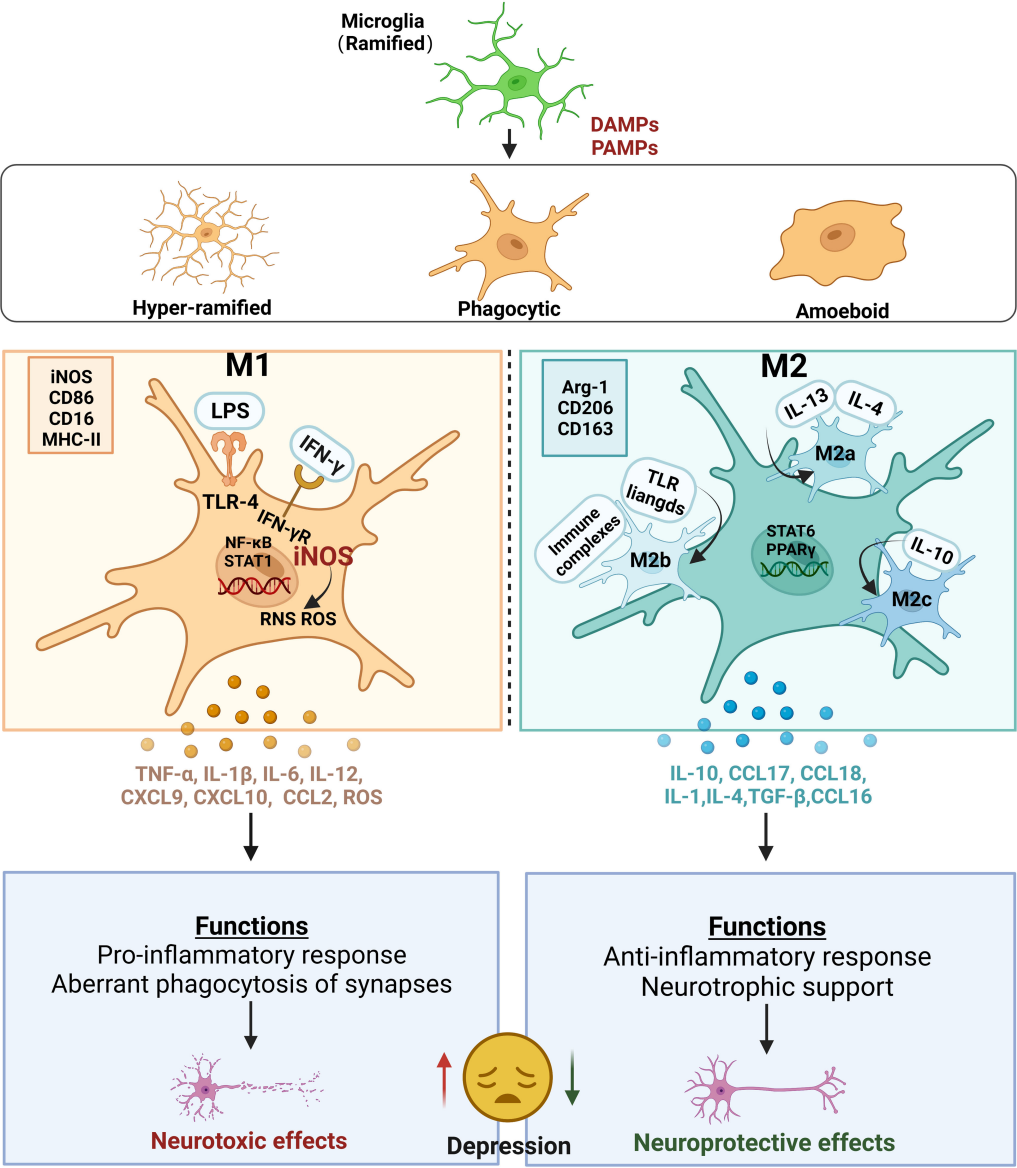
Microglial activation and their roles in depression. Ramified microglia stimulated by PAMPs or DAMPs are activated into three main microglial morphotypes, including hyper-ramified, activated, and amoeboid types. Alternatively, two main polarization states are defined in activated microglia: M1 and M2 phenotypes. The classic M1 polarization microglia is usually marked with iNOS, CD86, CD16 and MHC‐II. Under the M1 state, exposure to LPS and/or IFN‐γ stimulates and binds to TLR-4 or IFN‐γ receptors, respectively, leading to the activation of NF‐κB and STAT1. Meanwhile, the increase in iNOS produces a burst of ROS and RNS. All these lead to the excessive release of pro-inflammatory cytokines, such as TNF-α, IL-1β, IL-6, IL-12, and chemokines, such as CXCL9, CXCL10, CCL2, and ROS, which produce neurotoxic effects on the neurons in CNS. The alternative M2 polarization microglia is usually marked with ARG-1, CD206 and CD163. M2 microglia can also be divided into three subtypes including M2a, M2b and M2c, because of the different stimulis. Upon stimulation with IL‐4/IL‐13, the cells transform into the M2a-phenotype microglia with an increase in IL-10, CCL17 and CCL18. Exposed to TLR ligands and immune complexes, the cells transform into the M2b-phenotype microglia with an expression of IL-6. Upon stimulation with IL-10, the cells transform into the M2c-phenotype microglia with an increase in IL-4, TGF-β and CCL16. The phenotypes of M1 microglia perform functions that aberrant phagocytosis of synapses leading to produce neurotoxic effects on the neurons in CNS. The phenotypes of M2 microglia perform functions that neurotrophic support leading to produce neuroprotective effects on the neurons in CNS. Thus, the imbalance of M1 and M2 polarization induces increased excessive neurotoxicity and decreased neuroprotection, leading to depression. PAMPs pathogen-associated molecular patterns, DAMPs danger-associated molecular patterns, iNOS inducible nitric oxide synthase, CD86 cluster of differentiation 86, CD16 cluster of differentiation 16, MHC‐II major histocompatibility complex II, LPS lipopolysaccharide, IFN‐γ interferon gamma, TLR-4 toll-like receptor 4, NF‐κB transcription factors nuclear factor kappa-B, STAT1 signal transducer and activator of transcription 1, ROS reactive oxygen species, RNS reactive nitrogen species, TNF-α tumor necrosis factor α, IL-1β Interleukin-1β, Interleukin-6 IL-6, Interleukin-12 IL-12, CXCL9 chemokine (C-X-C motif) ligand 9, CXCL10 chemokine (C-X-C motif) ligand 10, CCL2 chemokines chemoattractant cytokine ligand 2, CNS central nervous system, ARG-1 arginase-1, CD206 cluster of differentiation 206, CD163 cluster of differentiation 163, IL‐4 interleukin-4, IL‐13 interleukin-13, IL-10 interleukin-10, CCL17 chemoattractant cytokine ligand 17, CCL18 chemoattractant cytokine ligand 18, IL-1 interleukin-1, Interleukin-10, IL-10 Interleukin-4 IL-4, TGF-β transforming growth factor-β, CCL16 chemoattractant cytokine ligand 16.

In many clinical reports, microglial activation in the insula, anterior cingulate cortex, and prefrontal cortex (PFC) have been found in patients with depression and those with suicidal ideation (67). These results suggest that the degree of microglial activation is positively correlated with depression severity (68). In rodents, chronic stress, including chronic unpredictable mild stress (CUMS), chronic social defeat stress (CSDS), and chronic restraint stress (CRS), commonly used to induce depression, results in elevated peripheral cytokines and the activation of microglia in stress-sensitive brain regions, such as the hippocampus, PFC, and amygdala (69). In contrast, minocycline, a potential anti-inflammatory agent, significantly blocks microglial activation and has been shown to contribute to improving depressive-like behaviors (70–72). Similarly, a previous study found that lipopolysaccharide (LPS)-induced inflammation, a classical microglia activation pathway that acts via the TLR4/nuclear factor-k-gene binding signaling pathway, increased the risk of depression in mice, which could be improved by the microglial depletion of PLX5622 or minocycline (70, 73). This indicates that microglial activation is strongly associated with the development of depression (**Figure 2**).

Many studies have revealed how the activation of microglia leads to the occurrence and development of depression. Firstly, it involves inflammation and polarization of microglia. Reassuringly, some preclinical studies have conducted more in-depth investigations in this area (74). Tang et al. found that CSDS mice showed higher M1 microglia markers, including iNOS, cluster of differentiation 16 (CD16), CD86, and C-X-C motif chemokine ligand 10, and no significant changes in M2 markers such as arginase-1 (Arg-1) and CD206 in the hippocampus at the transcriptional and protein levels, suggesting that M1 polarization plays a vital role in depression pathogenesis (75). Another study revealed that although no change in the total microglia number in CUMS-induced depressive mice was observed, much more activated microglia expressing M1-marker CD68 and less M2-marker CD206 was detected throughout the DG, CA1, and CA2 regions in the hippocampus (76). Taken together, the activation of microglia in susceptible brain regions towards the classical M1 polarization state instead of the M2 polarization state results in an increase in the production of toxic substances that harm neurons, which factors are crucial in the development and progression of depression (77).

Secondly, the phagocytic function of microglia in depression. The phagocytic function of microglia is considered an important factor affecting the occurrence and development of depression. Synapses interconnect neurons into networks that transmit neurotransmitters and mediate neuronal signaling, which contains pre-and postsynaptic separated by the synaptic cleft. Preclinical and clinical evidence suggests activated microglia in depressed animals and humans may aberrantly phagocytose neuronal synapses, resulting in synaptic dysfunction and depressive symptoms (78, 79). Preclinical studies in mouse models of depression have reported increased microglial phagocytosis of synapses in microglia-neuron contact areas (80). For example, there was a notable increase in the presence of phagocytic microglia actively engulfing puncta containing Postsynaptic density protein 95 (PSD95) in the DG of CSDS mice when compared with normal mice (81). Similar findings have been observed in other models, including decreased hippocampal expression of PSD95, synapses, and growth associated protein-43 and enhanced microglial phagocytosis in maternally separated rats. The hippocampus of chronic unpredictable mild stress mice also showed comparable results. Interestingly, intraperitoneal LPS injection in mice at postnatal day 50 to model early-life inflammation resulted in long-term phagocytosis of glutamatergic spines on neurons of the anterior cingulate cortex (82). The H3-receptor inverse agonist JNJ10181457 prevented abnormal LPS-induced microglial phagocytosis, decreased neuronal loss, improved neurotransmitter transmission, and had antidepressant effects in mice (83). Positron emission tomography revealed significantly increased translocator protein, a microglial marker, in the hippocampus, prefrontal cortex, and temporal cortex of depressed patients, suggesting microglial activation may contribute to depression pathophysiology. Functional imaging studies have also shown depressed patients exhibit decreased synaptic connectivity and neuronal circuitry in the prefrontal cortex and hippocampus (84). Together, these findings suggest aberrant phagocytosis of synapses by activated microglia in the hippocampus may underlie decreased synapses observed in depression. Therefore, in addition to modulating neuroinflammation, regulating microglial phagocytosis of synapses may provide novel insights into the mechanisms and therapeutic approaches for depression.

Finally, the trophic support function of microglia and endogenous microglial reduction is closely related to depression. Microglia support neuronal plasticity, neurogenesis, neural circuits and synaptic integrity through neuroimmune mechanisms including phagocytosis and cytokine secretion under baseline physiological conditions (85–87). Microglia also produce and respond to trophic factors like brain derived neurotrophic factor (BDNF) (88). BDNF was first isolated from the porcine brain in 1982 and found to support the survival of cultured embryonic sensory neurons (89). Considerable clinical evidence over subsequent decades has demonstrated BDNF plays an important role in depression (90). Regulating the C-X3-C motif chemokine receptor 1 (CX3CR1) in horses with phytoestrogens such as genistein increases BDNF and TGF-β secretion, promoting neuron-microglia interactions and synaptic remodeling. Endogenous microglial reduction or impairment may deprive neurons of adequate nutrition and energy, disrupting their function and contributing to depression (91). Kreisel et al. first reported the chronic unpredictable stress-induced microglial loss in the hippocampus, which could worsen severe depression (92). Administering innate immune stimulants like LPS and macrophage colony-stimulating factors to increase microglial numbers in the DG has reversed depressive behaviors in chronic stress mice. For instance, a single intraperitoneal 75 or 100 μg/kg dose of LPS reversed depressive behaviors of chronic unpredictable stress mice within 5 hours, as demonstrated by behavioral testing (93). The antidepressant effect of low-dose LPS depends on microglial activation regulating extracellular signal-regulated kinase (ERK) 1/2 signaling to increase BDNF synthesis. These findings suggest restoring endogenous microglial levels and function may benefit severe depression treatment, in addition to modulating microglia directly.

## 4 The function of microglia in adult hippocampal neurogenesis and its molecular mechanism in depression

Microglia are actively involved in the modulation of adult neurogenesis. Under physiological conditions, each of the individual components of adult neurogenesis, including the proliferation of NSCs, the migration of adult neuronal cells and immature neurons, their differentiation to mature neurons, and the extension of neuronal synapses, and integration of immature neurons into an existing circuit, is influenced by microglia (94, 95). First, microglia, as brain-resident immune cells, play a “housekeeping” role during neural development; that is, the proliferation of NSCs is controlled by microglia, and excess apoptotic proliferating cells are engulfed by microglia (49, 95, 96). Microglia release soluble factors that promote different stages of neurogenesis. It has been shown that nutrient factors such as insulin-like growth factor-1 (IGF-1) and brain-derived neurotrophic factor (BDNF), which are secreted by microglia, help the neural stem cell proliferation and the differentiation and guide the migration of neural precursor cells (97, 98). In addition, microglia modulate the integration of new neurons into existing neuronal circuits and refine neural circuits during development by pruning neuronal synapses (99). Therefore, microglia can participate in multiple stages of adult neurogenesis and play key roles in supporting adult neurogenesis.

**Figure 3:**
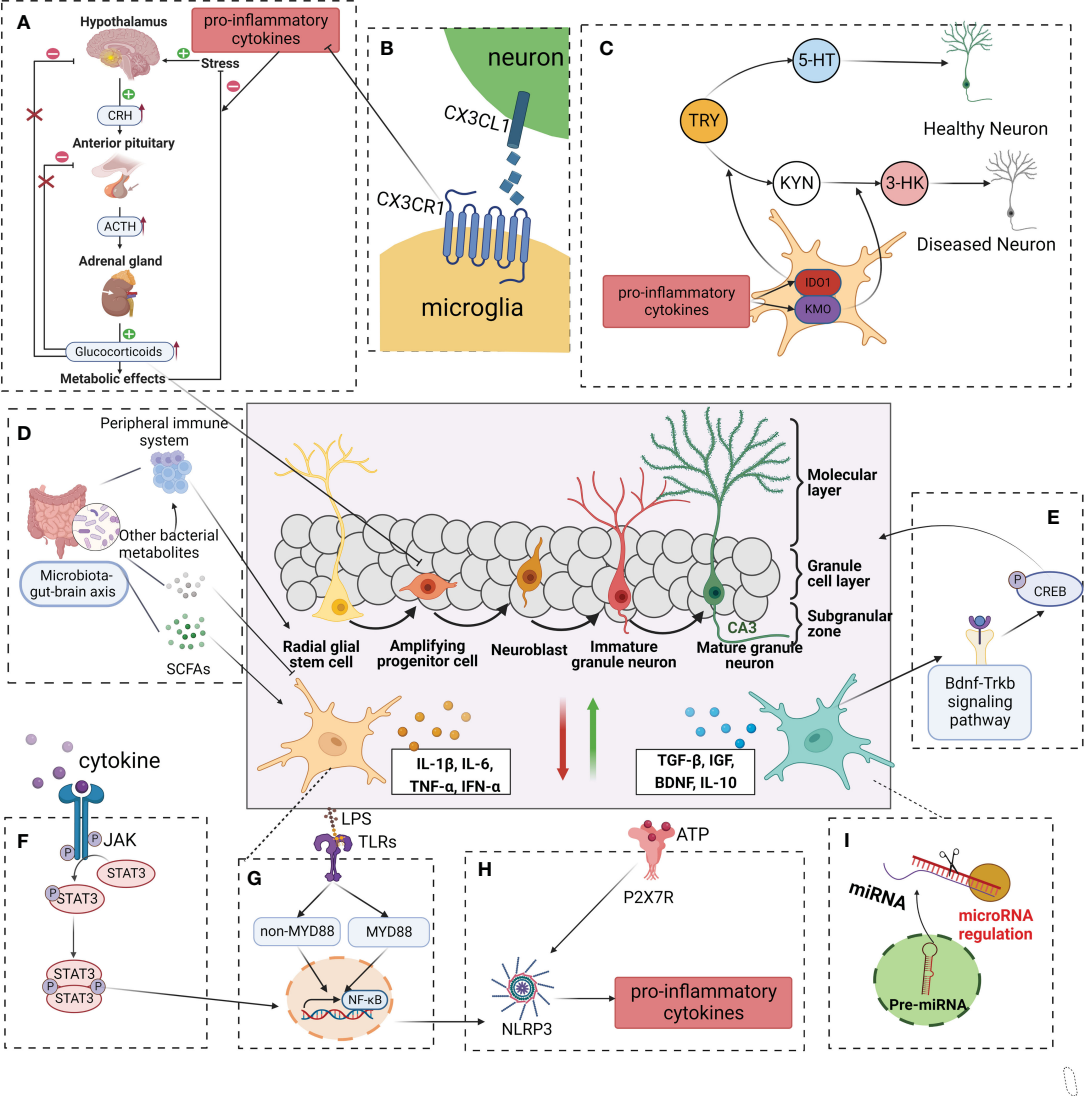
Modulation of adult neurogenesis by microglia in depressive. In a healthy central nervous system, microglia produce neurotrophic factors, phagocyte redundant neurons, and connections, remove cell debris, and control stem cell proliferation, in this way regulating synaptogenesis and neuronal pruning. In a pathological state, exogenous and endogenous factors such as infections(LPS), stress, and systemic inflammation/metabolic deregulation can induce microglial activation and impaired neurogenesis. **(A)** Cytokines such as IL-1β, IL-6, and TNF-α, with negative consequences to the HPA axis. **(B)** CX3CR1 is involved in interactions between microglia and neurons, which may reduce to pro-inflammation cytokine. **(C)** Inflammatory microglia(M1) also exhibit upregulated IDO, reducing serotonin availability and possibly contributing to neurogenesis in depression. **(D)** SCFAs、other bacterial metabolites and components of the immune system can affect microglial maturation, activation and function. **(E)** A neuroprotective microglial phenotype (classically referred to as “M2-like”) activated the BDNF-TrkB-CREB signaling pathway, enhanced neurogenesis, diminished synapse loss in the hippocampus, and contribute to an overall lower level of neuroinflammation. **(F–H)** Intracellularly, several pathways become activated including the TLR, JAK, and STAT, which will trigger the activation of the NF-κB, downregulate NLRP3, and consequent induction of first-line cytokine production, such as IL-1β, IL-6, and TNF-α. **(I)** MicroRNAs have been associated with multiple pathways of depression pathophysiology. LPS lipopolysaccharide, TLR toll-like receptor, JAK Janus kinase, STAT signal transducer and activator of transcription, NF-κB nuclear transcription factor-kappa B, NLRP3 nod-like receptor protein 3, IL-1β interleukin-1β, IL-6 interleukin 6, TNF-α tumor necrosis factor-alpha, HPA hypothalamic-pituitary-adrenal, IDO indoleamine 2,3-dioxygenase, CX3CR1 C-X3-C motif chemokine receptor 1, BDNF- TrkB-CREB brain derived neurotrophic factor- tyrosine kinase receptor B- cyclic adenosine monophosphate response element binding protein, SCFAs short chain fatty acids.

There are many existing models of depression, and different models simulate different types of depression with distinct internal mechanisms. Currently, reports have focused on models involving the microglia mechanisms, neurogenesis mechanisms, or microglia-neuron regulation of neurogenesis mechanisms. These models focus on early life stress (maternal separation (MS), maternal sleep deprivation, and adult stress (CUMS and chronic mild stress (CMS)), as well as toxic infection (LPS) models. Additionally, genetic mutations have also been reported to cause depression (**Table 1**). Under pathological conditions, microglia can contribute to depression through a variety of mechanisms, including toll-like receptors (TLRs), nod-like receptor protein 3 (NLRP3) inflammasome, lesion of the HPA axis, metabolism of 5-hydroxytryptamine (5-HT), inflammatory cytokines, and their mediated inflammatory signal pathways, imbalance of M1/M2 polarization, gut microbiota, and microRNA. These mechanisms are described in further detail below (**Figure 3**).

**Table 1:** Expression patterns of depression-like behaviors, microglia, inflammatory molecules, and molecular markers of neurogenesis in rodent models of depression.

Depression model	Stress	Animal	Test/Score	Behavioral Effects	Microglia	Pro-inflammation	Antiinflammation	Proliferation	Differentiation	Reference
Early-life stress	MS for 14 days	♂ C57BL/6 J mice(baby)	SPT FST OFT	control and immobility time >mean of control The SPT and OFT showed no significant differences between maternal-separated or control mice in terms of the number of spontaneous movements	↑Activated microglia ↓The average number and length of microglial processes ↓Expression of the microglial marker CX3CR1	↑iNOS ↑TNF-α ↑IFN-γ ↑IL-6 ↑IL-1β	↓IL-4 ↓TGF-β ↓IL-1rα ↓Ym-1 ↓Arg-1	↓BrdU^+^	↓BrdU^+^/DCX^+^	(100)
Adult stress	CUMS for 4 weeks	♂ Sprague Dawley rats (3 weeks)	SPT OFT MWM	↓sucrose preference ↓Time in the center of the open field ↑escape distance	↑the number of Iba-1^+^ microglia ↓the mean number of intersections and surface area ↓ramification length ↑soma volume	——	——	——	↓DCX^+^	(101)
	CUMS for 21 days	Cx3cr1CreERT2 mice and Nr3c1fl/fl mice (6.5 to 8.5 weeks)	Saccharin preference PR	↓Body weight ↓Saccharin preference	↑arborization area ↑Cell body areaTrem2 ↑Cx3cr1 ↑Mertk	↑CD68 ↑TNF-α ↑IL-1β	↓Arg-1 ↓Fizz-1	↓Ki-67	↓DCX	(102)
	CUS for 5 weeks	♂ C57BL/6 mice (3-4 months)	SPT NOR	↓Exploration time Sucrose preference	↓Microglia number ↓Processes length ↑Soma area	——	——	——	↓DCX^+^	(103)
	CUS for 6 weeks	♀ Sprague-Dawley rats (10 months)	SPT NSF FST	↑immobility and swimming and struggling behavior in FST ↑anxiety-like behavior in NSF ↑anhedonia-like behavior in the SPT ↓Body weight	the number and length of Iba-1^+^ microglia are equal in OVX and sham surgery	——	——	——	——	(104)
	CMS for 3 weeks	♂ C57BL/6J mice (8-10 weeks)	TST FST	↑immobility and swimming and struggling behavior in FST ↓sucrose preference	↓Microgia number ↓Processes length ↑Soma area ↑iba -1^+^	↑iNOS ↑CD86 ↑CD45 ↑CD11b	↓IL-4 ↓Arg-1 ↓CD206	↓BrdU^+^	↓BrdU^+^/DCX^+^	(105)
Infectious organisms	0.83 mg/kg LPS(I.p.)	♂ ICR mice (8 weeks)	SPT OFT TST FST	↑immobility time in the FST and TST ↓sucrose preference	↑the number of Iba-1^+^ microglia ↓the number of ramifications	↑IL-6 ↑TNF-α ↑IL-18	↑IL-4 ↑IL-10	——	↓DCX^+^	(106)
	0.83 mg/kg LPS(I.p.)	♂ ICR mice	SPT OFT TST FST	↓the total distance traveled ↓the time spent in the central area ↓the inner area distance traveled ↓sucrose preference in the SPT ↑immobility time in the TST and FST	↑the number of Iba-1^+^ microglia ↓the number of ramifications	↑CD68 ↑CD16 ↑CXCL10 ↑iNOS ↑IL-1β ↑TNF-α ↑IL-18	↑Arg-1 ↑CD206	——	↓DCX^+^	(107)
Gene mutation	——	♂ Arid1afl/fl mouse(1-2 weeks)	OFT LDB EPM MWM FC MBT Self-grooming test	↓Body weight ↓the time in the center of the open field ↓the time In the light region The open arm is less significant in the elevated crypto,did not cause any difference in Y maze experiment and MWM	↑Soma area ↑iba-1	——	↑Arg-1	↓BrdU^+^	↓NeuN^+^BrdU^+^ ↓DCX^+^Brdu^+^	(108)

BrdU, 5-Bromo-2-deoxyuridine; CD, Cluster of differentiation; CMS, Chronic mild stress; CUMS, Chronic unpredictable mild stress; CUS, Chronic unpredictable stress; CX3CR1, C-X3-C motif chemokine receptor 1; EPM, Elevated plus maze; FC, Fear conditioning; FST, Forced swimming test; Iba-1, Ionized calcium binding adapter molecule 1; IL, Interleukin; IFN-γ, Interferon-γ; I.P., Intraperitoneal injections; iNOS, Inducible nitric oxide synthase; LDB, The light–dark box; MBT, Marble burying test; MS, Maternal separation; MWM, Morris water maze; NOR, Novel object recognition; NSF, Novelty-suppressed feeding test; OFT, Open field test; PR, Progressive ratio schedule; SPT, Sucrose preference test; TGF-β, Transforming growth factor-β; TNF-α, Tumor necrosis factor α; TST, Tail suspension test.

### 4.1 TLRs activate microglia to reduce neurogenesis

TLRs, expressed on microglia are a subfamily of pattern recognition receptors (PRRs) that identify endogenous noxious stimuli and invading pathogens, thereby inducing induction of innate and adaptive immune responses (109, 110). Experimental results demonstrated that social avoidance and anxiety induced by repeated social defeat stress (R-SDS) were abolished in mice lacking Toll-like receptors 2 and 4 (TLR2/4). Transcriptome analysis further revealed that microglia in the brains of R-SDS mice induced the expression of IL-1α and TNF-α via the TLR2/4 signaling pathway. Treatment capable of blocking microglial-mediated cytokine release was able to attenuate R-SDS-induced social avoidance. These results suggest a crucial role of TLR2/4 in microglial activation and cytokine release in response to R-SDS-induced stress (111). Research has revealed that Toll-like receptor 5 (TLR5) expression in mouse embryonic stem cells promotes neurodifferentiation and enhances it through the nuclear factor-κB and interleukin 6/CREB pathways. Furthermore, TLR5 expression was also observed in the SGZ of the hippocampus in rat, where it governs the proliferation of adult hippocampal NSCs by regulating the cell cycle, and promotes neural differentiation through the JNK pathway. These findings provide evidence of TLR5’s significant modulatory role in neurogenesis from embryonic stem cells and adult hippocampal NSCs, and suggest it is a promising therapeutic target for Brain-related diseases (112). In contrast, promising immunosuppressive drugs, such as icariin, can improve neuroinflammation in the hippocampus by inhibiting the activation of the TLR4-NF-κB signaling pathway to exert protective effects on neurogenesis, thereby improving depressive behavior in mice (113). These results provide a new understanding of depression pathology, wherein inflammation can be triggered by activating TLRs in microglia, resulting in a reduction in neurogenesis, and ultimately playing a crucial role in the development of depression.

### 4.2 NLRP3 inflammasome triggers microglia to regulate neurogenesis

Inflammasomes are cytosolic immune signaling complexes that lead to inflammation and pyroptosis. They are composed of pattern recognition receptors, adapter structural domains and enzymatic caspase-1 structural domains. Inflammasome initiation sensors are PRRs, including nucleotide-binding oligomerization domains leucine-rich repeat-containing protein receptors (NLRs), which are not present in melanoma-2-like receptors, and proteins with tripartite motifs (114). NLRs consist of NLRP1, NLRP2, NLRP3, NLRP6, NLRC4, and NLRP12, of which NLRP3 has received the most attention in studies of the pathological mechanism of depression (115, 116). Male C57BL/6 mice were subjected to CUMS for a period of 6 weeks. A comprehensive assessment of negative emotional behaviors was carried out to determine the susceptibility of the mice. Various parameters such as microglial activation, transcription of endogenous retroviral sequences, intrinsic nucleic acid sensing response, and immune inflammation were evaluated in the Basolateral Amygdala. The findings of the study revealed that chronically stressed mice exhibited significant depressive and anxious-like behaviors, accompanied by notable microglial cell morphological activation, transcription of MuERV-L, MusD, and IAP genes, activation of the cGAS-IFI16-STING pathway, initiation of the NF-κB signaling pathway, and activation of the NLRP3 inflammasome in the Basolateral Amygdala (115). However, these abnormal indicators were gradually reversed after antidepressant administration, suggesting a close link between depression and the NLRP3 inflammasome. For example, Du et al. found that fluoxetine, a classical antidepressant, can remarkably suppress the activation of NLRP3 inflammasome, followed by reduced caspase-1 cleavage and IL-1β which were secreted in peripheral macrophages and central microglia (117). Recent studies have demonstrated that they can diminish the expression of NLRP3 inflammasome and lower the levels of inflammatory factors that are regulated by microglia, ultimately providing anti-depressant effects. NLRP3 is also involved in the regulation of neurogenesis. Many studies have shown that microglia triggered by NLRP3 inflammasome activation secrete many more pro-inflammatory cytokines, such as IL-1β, thus resulting in the reduction of hippocampal neurogenesis (118). Conversely, mice were exposed to CMS for three weeks, and the results revealed that CMS exposure altered the morphology of microglia in the hippocampus and increased the number of TUNEL^+^ and GSDMD^+^ microglia in the dentate gyrus (119). Furthermore, the CMS exposure led to a decrease in the number of 5-ethynyl-2-deoxyuridine (BrdU) ^+^, BrdU-DCX^+^, and BrdU-NeuN^+^ cells in the hippocampal dentate gyrus, as well as a reduction in the percentage of NeuN^+^ cells among BrdU^+^ cells. Additionally, the expression of pyroptosis-related molecules, such as NLRP3, caspase-1, GSDMD-N, and IL-1β, was significantly upregulated in the hippocampus of CMS-exposed mice (119). These findings indicate that CMS exposure impairs hippocampal neurogenesis and induces microglial pyroptosis. The study found that porphyran suppressed the NF-κB/NLRP3 signaling pathway in the hippocampus and activated the BDNF/TrkB/ERK/CREB signaling pathway in CUMS mice, thereby inhibiting the activation of microglia and reducing the release of pro-inflammatory cytokines mediated by microglia (120). It also increased the expression of DCX in the hippocampal DG area, promoted neurogenesis, and maintained synaptic plasticity, ultimately exerting an antidepressant effect (120). These data have shown that NLRP3 plays a key role in the regulation of neurogenesis, a key molecular mechanism in the pathogenesis of depression.

### 4.3 Imbalance of polarization of microglia affect neurogenesis

The M1 and M2 polarization of microglia differentially regulates neurogenesis in physiological and pathological states (121). M1 polarization of microglia is negatively correlated with neurogenesis (117). The M1 phenotype of microglia secretes excessive inflammatory mediators that lead to impaired neurogenesis and promote the onset and progression of depression. Additionally, pro-inflammatory mediators such as macrophage inflammatory protein-2 (MIP2), IL-1β, iNOS, and TNF-α activate M1 microglia, which then produce many more damaging factors that reduce hippocampal neurogenesis and induce depression (121). The activation of microglia into the M1 state affects neurogenesis by inhibiting the proliferation of NSCs, increasing the apoptosis of neural progenitors, and reducing the survival of newborn neurons and their integration into existing neural circuits (122). In contrast, M2 polarization of microglia, an alternative subtype of microglial activation, is positively correlated with neurogenesis. A recent study found that the alternatively activated microglial phenotype (M2), which is activated by IL-4, enhances neurogenesis and thus ameliorates depression-like behaviors (123). Therefore, modulating the microglial phenotype to regulate neuroinflammation and neurogenesis appears to be a possible mechanism for the development of depression.

### 4.4 Transcription (STAT) family members activate microglia to regulate neurogenesis

The Janus kinase (JAK)/STAT pathway is engaged in the modulation of a number of CNS functions, involving neurogenesis, synaptic plasticity and microglia activation, all of which are implicated in the pathophysiology of depression (124, 125). First, JAK/STAT signaling is important for regulating inflammatory responses and microglial polarization (125). Studies have shown that inhibition of the JAK/STAT1 pathway can block microglial activation and suppress microglia-mediated neuroinflammation. Kwon et al. found that microglia-specific STAT3 KO mice showed antidepressive-like behavior in the animal model of chronic stress procedures, suggesting that suppression of microglia STAT3 may be a new treatment tactic for depression (126). Furthermore, recent studies have indicated that JAKs may have a role in hippocampal neurogenesis. For instance, research has demonstrated that pharmacologically inhibiting JAK3 in cultured neurons can stimulate neurogenesis (127). Other studies have shown that JAK3 is involved in inhibiting neurogenesis and causing depressive symptoms in animal models under stress (128, 129). As a result, JAK3 inhibition has the potential to improve depressive and anxious behavior and restore normal levels of hippocampal neurogenesis in mice subjected to stress (130). However, microglia modulate the mechanisms of microglia as well as neurogenesis in response to depression via the JAK/STAT signaling pathway is unclear. Therefore, it is important to concentrate on the JAK/STAT signaling pathway in microglia and elucidate its relationship with depression. Such research may provide new insights for future studies on depression.

### 4.5 Activation of Fractalkine-CX3CR1 in microglia suppresses neurogenesis

The signaling pathway of Fractalkine-CX3CR1 is essential for regulating of interactions between microglia and neurons (131). Fractalkines, also called CX3CL1, are transmembrane chemokines expressed by neurons in the brain. It reduces migration and increases neuronal adhesion (132). A combination of CX3CR1 and CX3CL1, which is only expressed by microglia, maintains microglia in a quiescent state, and hence inhibits the release of pro-inflammatory cytokines (133). CX3CR1 is significantly upregulated in depression (134). The character of microglia in modulating neurogenesis in depression via the CX3CL1-CX3CR1 signal is complex and controversial. Evidence has shown that CX3CL1-CX3CR1 signaling participates in the control of microglial activation, and thus influences adult neurogenesis. For instance, there are reports indicating that compared with wild-type mice, the number of DCX^+^ positive cells and BrdU^+^ labeled cells in the hippocampus of CXCR1 deficient mice is reduced, indicating that neurogenesis is inhibited due to CX3CR1 deficiency (135). Similarly, studies have shown that the deficiency of the CX3CR1 of microglial damages synaptic integration in hippocampal granule neurons in adult neurogenesis, promotes the activation of microglia in the DG of CXCR1 deficient mice, and induces a pro-inflammatory phenotype in this area of DG (136). These results suggest that microglia regulate adult hippocampal neurogenesis via CX3CL1-CX3CR1 signaling. However, many contradictory reports have been published. For example, Mattison et al. found that the freeing of pro-inflammatory factors such as IL-6 was significantly reduced by LPS administration in CX3CR1 knockout mice, suggesting an obvious suppression of the inflammatory response in CX3CR1 knockout mice (137). Moreover, Liu et al. found that deficiency of CX3CR1 might promote alternative activation of microglia (M2 polarization) and attenuate their ability to synthesize and release inflammatory cytokines, thus benefiting adult neurogenesis (138). Therefore, the role of CX3CL1/CX3CL1 signaling in microglial activation and subsequent regulation of neurogenesis in depression is an intricate and controversial topic of research, which is further complicated as new studies have revealed inconsistent results.

### 4.6 Abnormalities in the HPA axis inhibit adult neurogenesis involving pro-inflammatory cytokines released by microglia

The HPA axis is a complicated combination of interactions concerning the pituitary, hypothalamus gland, and adrenal gland. The HPA axis is an essential component of the neuroendocrine system and is related to the control of stressful responses and the pathophysiology of a lot of neuropsychiatric disorders (139). Inflammation and stress stimulate the hypothalamus to secrete corticotropin-releasing hormone, which further stimulates the pituitary to release corticotropin. The adrenal gland releases glucocorticoids, which can act on the hypothalamus for negative feedback regulation. The HPA axis is hyperactive during depression (140). Abnormalities in the HPA axis are observed in a substantial proportion of patients with depression and are primarily characterized by glucocorticoid dysregulation (141). Studies have shown that excessive glucocorticoids reduce the proliferation of nerve cells, thereby inhibiting adult neurogenesis (142). Moreover, adult hippocampal neurogenesis can mediate negative feedback through the HPA axis. Snyder et al. used the spatial specificity of X-ray irradiation to reduce hippocampal neurogenesis, resulting in excessive secretion of glucocorticoids (135). Taken together, glucocorticoids reduce adult hippocampal neurogenesis, resulting in the failure of negative feedback that is critical for the HPA axis, which leads to a persistent elevation of glucocorticoids and reduces adult hippocampal neurogenesis. This vicious cycle may play an essential character in depression pathophysiology. Interestingly, regulation of the HPA axis is also influenced by cytokines released by microglia. For example, microglia-discharged pro-inflammatory cytokines, such as IL-1 and IL-6, can effectively induce glucocorticoid secretion during chronic and acute stress (143). Therefore, microglia may regulate the HPA axis by releasing pro-inflammatory factors and elevating glucocorticoids to reduce adult hippocampal neurogenesis, thereby playing a crucial role in depression.

### 4.7 Reduction of 5-HT suppresses neurogenesis relates to the activation of microglia

TRY metabolism involves two important metabolic pathways: 5-HT synthesis and kynurenine synthesis. The 5-HT synthesis is initiated by tryptophan hydroxylase, which converts TRY to hypoxanthine, which in turn is converted to 5-HT by serotonin synthetase (144). It is currently known that 5-HT strongly correlates with the pathogenesis of depression. Selective serotonin reuptake inhibitors (SSRIs), the most common clinical antidepressants, improve depressive mood by adding the 5-HT levels of the synaptic cleft (145). Studies have reported that 5-HT is synthesized by 5-HT neurons located in the raphe nucleus, and has significant implications in the regulation of neurogenesis. Removal of 5-HT neurons in the dorsal raphe and median raphe has been found to decrease neurogenesis, which is recovered by 5-HT reinnervation. Increased 5-HT levels have been shown to promote neurogenesis, thereby improving depressive behavior (146). Thus, the depletion of 5-HT suppresses neurogenesis in the DG, which is a hypothesis for the pathogenesis of depression. Another catabolic pathway of TRY contains indoleamine 2,3-dioxygenase (IDO) and TRY 2,3-dioxygenase (TDO), which produce by metabolism from TRY to kynurenine (KYN) (147). When the KYN metabolic pathway is enhanced, it deprives TRY of 5-HT synthesis, resulting in reduced serotonin production (148). Increased activation of the KYN pathway has been observed in depressed patients, and the level of activation is relevant to the level of depression (149). KYN can be further metabolized into several neurotoxins such as 3-hydroxykynurenine (3-HK), which is catalyzed by kynurenine monooxygenase (KMO) (150). Numerous studies have shown that pro-inflammatory cytokines stimulate IDO and KMO, which are mainly expressed in microglia, thereby increasing neurotoxins, which is one of the potential mechanisms by which inflammation is mediated by microglia and induces depression (150). However, the mechanism by which microglial activity interacts with KYN metabolites has not been definitively determined, though we can infer that under stress conditions, pro-inflammatory factors released by activated microglia activate the kynurenine pathway, resulting in a decrease in the synthesis of 5-HT and an increase in neurotoxins such as 3-HK, which may inhibit neurogenesis and lead to depression.

### 4.8 Cytokines secreted by microglia participate in neurogenesis

Cytokines are redundant secreted proteins with growth, differentiation, and activation functions that regulate and determine the nature of immune responses and control immune cell trafficking and cellular arrangement of immune organs (151). Inflammatory cytokines consist of pro-inflammatory and anti-inflammatory cytokines, the balance of which determines whether the body produces an inflammatory response. The role of cytokines in neurogenesis is reflected in the fact that pro-inflammatory cytokines impair neurogenesis, whereas anti-inflammatory cytokines protect or promote neurogenesis. Excessive pro-inflammatory cytokines induce an inflammatory response, which can induce the expression and release of cathepsin C in microglia to promote an inflammatory response and reduce neurogenesis (152). For example, IL-1β, IL-6, TNF-α, and IFN-α are currently the broadest studied inflammatory cytokines (153). Multiple studies have suggested that TNF-α and IL-6 levels are increased remarkably in the peripheral blood of depressed patients than in healthy controls, suggesting a strong association between inflammatory cytokines and depression (153). This mechanism may be related to the activated M1 microglial phenotype (154, 155). Conversely, anti-inflammatory cytokines, including TGF-β and IL-10, secreted by M2 phenotype microglia, exerted anti-inflammatory effects. Furthermore, existing evidence suggests that inflammatory cytokines are strong relativity of neurogenesis. For instance, CMS exposure in mice was found to upregulate inflammatory factors and inhibit neuronal growth in the dentate gyrus region of the hippocampus, leading to depression-like behavior (156). Surprisingly, drug treatment reprogrammed Arg-1^+^ microglial cell phenotypes in the dentate gyrus region, suppressed neuronal inflammation, increased the quantification of hippocampal newborn neurons (BrdU^+^ - DCX^+^ cells), and improved depression-like behavior in CMS-exposed mice (156). Thus, the balance of cytokines participates in neurogenesis, thus influencing the occurrence of depression.

### 4.9 BDNF and its cascade signaling promote neurogenesis associated with microglia

BDNF, a protein synthesized in the brain and are widespread in the CNS, has critical function in the survival, differentiation, growth, and development of neurons (157). Tyrosine kinase receptor B (TrkB) acts as a BDNF specific receptor. Binding to the TrkB receptor activates intracellular signaling pathways that play an important role in sustaining neural growth, neuronal differentiation, and neuronal survival, as well as in maintaining synaptic plasticity and neuronal structure and function in adults (158). Wen et al. found that BDNF and TrkB were significantly reduced in the brain, especially in the hippocampus of mice, with significantly increased depressive and anxiety-like behaviors, suggesting a strong link between depression and the BDNF-TrkB signaling pathway (159). This mechanism may be related to the function of the BDNF-TrKB signaling pathway in neurogenesis. For example, Sonoyama et al. demonstrated that BDNF variants and variants of the TrkB kinase domain can lead to damaged processing and secretion of mature peptides in the BDNF-TrKB signaling pathway, resulting in impaired neurite growth and inhibition of hippocampal neurogenesis (160). Notably, Li et al. have shown that upregulation of the BDNF-TrkB signaling pathway can promote neuronal plasticity and thus increase the antidepressant response (161). In addition, some studies have found that the BDNF-TrkB signaling pathway is associated with microglia. Bagheri et al. found that high activation of M2 microglia and decreased activation of M1 microglia resulted in increased BDNF secretion (162). Ding found that BDNF can accelerate the activation of microglia to free TNF-α and IL-1β, aggravating the neuroinflammatory response through the BDNF-TrkB signaling pathway (162). The microglial subset with high expression of Arg-1 driven by IL-4 is crucial for maintaining hippocampal neurogenesis and stress resilience. In a mouse model, reducing Arg-1^+^ microglia led to decreased secretion of BDNF from Arg-1^+^ microglia in the DG region of the hippocampus, resulting in reduced survival and maturation of NSPCs and inhibition of hippocampal neurogenesis, as well as increased susceptibility to stress-induced depression. Conversely, enhancing IL-4 signaling to increase Arg-1^+^ microglia restored hippocampal neurogenesis and resistance to stress-induced depression (105). Therefore, microglia can promote neurogenesis and reduce depressive symptoms by acting on the BDNF or BDNF-TrkB signaling pathway.

### 4.10 Microbial gut-brain axis regulates the crosstalk between microglia and neurogenesis

The gut-brain axis is a two-way relationship between the gut and CNS connected by neurons of the sympathetic and parasympathetic nervous systems, that has been shown to induce stress-related gastrointestinal and mental symptoms. The gut microbiome is strongly connected to the gut, and by extension the brain, thus extending the gut-brain axis to the gut-brain-microbiota axis (163). Recently, dysregulation of the gut-brain-microbiota axis has been emerged as one of the leading hypotheses for explaining the pathogenesis of depression (164). Yu Du. found that mouse treated with antibiotics demonstrated a reduction in saccharin preference due to more than 90% of the gut microbiota being killed by antibiotics, which may indicate the key function of the gut microbiota in depressive-like behaviors (164). The underlying mechanism may be related to the significant role of gut microbiota in regulating the maturation and activation of microglia. Sun et al. found that changes in gut microbiota led to systemic inflammation that differentially activated inflammatory regulatory pathways in the brain, especially those associated with microglia, leading to the occurrence of depression (165). Carlessi et al. found that gut microbes secrete substantial amounts of microglia-activated amyloid and LPS to induce depression (166). Furthermore, several clinical and experimental studies have indicated that gut microbiota may be both a pathogenic determinant of neurogenesis-related diseases and a novel therapeutic target. For example, Sarubbo et al. found that germ-free (GF) and GF-colonized mice exhibited a trend of cell proliferation with elevated expression of BrdU in the dorsal hippocampus (167). Notably, Cerdó et al. found that using a strain-specific combination of probiotics restores neurogenesis defects in adult patients, which further confirms the link between gut microbiota and hippocampal neurogenesis (168). Previously, literature reported that regulating short-chain fatty acids, which are widely present in intestinal endocrine and immune cells and are important mediators for regulating body function through the gut microbiota, can decrease the ratio of Iba-1^+^/CD68^+^ cells in the hippocampal DG area of CUMS mice while increasing Ki67/NeuN, granular cell layer width, and dendritic spine density and quantity of neurons, thereby promoting neurogenesis and exerting an anti-depressant effect (101). In addition, Hanna Karakuła-Juchnowicz et al. found that gut dysbiosis and irritation may dysregulate the immune system near the brain, causing neurodegeneration (169). These results suggest that the microbial gut-brain axis may influence microglial activation and neurogenesis in depression. However, the function of the microbial-gut-brain axis in microglial regulation of neurogenesis requires further study.

### 4.11 MicroRNA regulates the link between microglia and neurogenesis

MicroRNA (miRNA) is a non-coding RNA (ncRNA) that consists of 18-24 nucleotides, which is highly conserved in species that modulate gene expression, mainly by destabilizing the target mRNA and inhibiting the translation of the target mRNA to regulate target mRNAs (170). Microglia utilize exosomes to transfer microRNAs, specifically miR-146a-5p, to inhibit neurogenesis in cases of depression. Overexpression of miR-146a-5p in the hippocampal DG leads to a suppression of excitatory neuron spontaneous discharge and neurogenesis via direct targeting of Krüppel-like factor 4 (KLF4). Downregulation of miR-146a-5p expression results in improved adult neurogenesis in the DG and alleviates depression-like behaviors in rats. Notably, circular RNA ANKS1B acts as a miRNA sponge for miR-146a-5p and mediates post-transcriptional regulation of KLF4 expression. These findings demonstrate the critical role of miR-146a-5p in regulating neurogenesis during pathological processes related to depression and indicate that exosomes from microglia provide a new communication pathway between glial cells and neurons (171). These data demonstrate that multiple miRNA genes in the human body have different effects on depression and neurogenesis, which will be further clarified. It was also found that different miRNAs also have different effects on the polarization of microglia (172). For example, neuron-derived exosomes with high miR-21-5p expression promote M1 polarization of microglia. In contrast, MiR-124-enriched exosomes promoted M2 polarization of microglia (173). Currently, there are few studies on the roles of miRNAs in the regulation of microglia and neurogenesis, despite the complex link between neurogenesis and microglia in depression. Thus, elucidating the regulatory mechanisms of miRNAs in depression from the perspective of the regulation of microglia in neurogenesis may provide new insights into depression.

## 5 Drugs targeting microglia to alter neurogenesis for treating depression

According to the above summary, microglia play a significant role in modulating neurogenesis and, thus, play a key function in the pathogenesis of depression. Therefore, here we introduced some drugs that act on this mechanism, some of which are already in clinical use, such as tricyclic antidepressants (TCAs) and SSRI, and some are under development, such as minocycline, ketamine, and natural products derived from plants (**Table 2**).

**Table 2:** Drugs targeting microglia to alter neurogenesis for treating depression.

Reference	Type	Drug	Model	Modeling method	Dosing	Mechanism: microglia/Neurogenesis/microglia -mediated regulation of neurogenesis.
(174)	First-line antidepressant drugs in clinical practice	clomipramine and imipramine	BV2 cells/primary microglia	treated with or without LPS (100 ng/ml) for 24 h	pretreated with Clomipramine (15 μM) and imipramine(10 μM) for 24h	activation microglia (indirectly)
(175)		imipramine	mice	RSD	oral (15 mg/kg) for 1 week	activation microglia (indirectly)
(176)		clomipramine	mice	injected with LPS (1 mg/kg)	I. p (20 mg/kg) for 1 week	activation.microglia (indirectly)
(177)		imipramine	mice	CUS for 5 weeks	oral (20 mg/kg)	microglia -mediated regulation of neurogenesis(directly)
		sertraline	mice	CUMS for 5 weeks	oral (5 mg/kg) for 5 weeks	activation microglia (indirectly)
(178)		fluoxetine	mice	UCMS for 6 weeks	oral (15 mg/kg) for 6 weeks	microglia -mediated regulation of neurogenesis(directly)
(100)	Potential anti-inflammatory candidate drugs	minocycline	mice	MS	I. p (20 mg/kg) for 2 weeks	microglia -mediated regulation of neurogenesis(directly)
(179)		ketamine	mice	Genetically engineered mouse	I. p (7 mg/kg)	regulating neurogenesis(indirectly)
(180)		ketamine	rat	treated with long-term corticosterone administration for 4 weeks	I. p (2.5, 5, 10 mg/kg)	regulating neurogenesis(indirectly)
(181)	Natural compounds	Xanthoceraside	mice	CUMS	I. p (0.02, 0.32 mg/kg)	regulating neurogenesis(indirectly)
(182)		Xanthoceraside	microglia	induction with Ab25–35 (10 mM)/IFN-g (10 U/mL) for 24-h	pretreatment (0, 0.001, 0.01, or 0.1 mM) for 16h	regulating neurogenesis(indirectly)
(183)		Curcumin	BV2 cells	treated with or without LPS (1 μg/mL) for 24 h	pretreatment (1,5, 10, 20, and 40 μM) for 2 h	regulating microglia (indirectly)
(184)		Paeoniflorin	mice	I. p of RESP (1 mg/kg) for 3 days	I. g (10, 20, 40mg/kg) for 3 days	regulating microglia (indirectly)
(185)		Paeoniflorin	mice	CUMS for 5 weeks	I. p (20 mg/kg) for 3 weeks	regulating neurogenesis(indirectly)
(186)		Paeoniflorin	rat	MCAO procedures	I. p (5, 10 mg/kg) for 2 weeks	microglia -mediated regulation of neurogenesis(directly)
(187)		Paeoniflorin	rat	permanent four-vessel occlusion	oral (40 mg/kg) for 4 weeks	microglia -mediated regulation of neurogenesis(directly)
(188)		Resveratrol	mice	I. p of LPS (1 mg/kg)	oral (30 mg/kg) for 7 days	regulating microglia (indirectly)
(189)		Resveratrol	microglia	treated with LPS (1 μg/ml) for 4 h and ATP (5 mM) for 1 h	pretreatment (10 µM) for 1h	regulating microglia (indirectly)
(190)		Resveratrol	mice	CLP	I. p (10, 30 mg/kg) at 1 hour prior to surgery and again at 6 h, 12 h, and 18 h after surgery	regulating microglia (indirectly)
(191)		Resveratrol	mice	I. p of LPS (5 mg/kg)	I. p (20 mg/kg) for 2 weeks	microglia -mediated regulation of neurogenesis(directly)
(192)		Resveratrol	mice	injected with 120 mg kg-1 of D-galactose (0.2 mL per 10/d) for 62 days	oral (functional fermented milk: 0.9% NaCl = 1: 3, functional fermented milk: 0.9% NaCl = 1: 1, functional fermented milk) for 62 days	regulating neurogenesis(indirectly)
(193)	Others	Melatonin	mice	I. p of LPS (5 mg/kg)	I. p (30 mg/kg) for 4 times	regulating microglia (indirectly)
(194)		Melatonin	rat	exposed to pyridostigmine bromide, DEET, and permethrin, and 15 min of restraint stress for 4 weeks	oral (5, 10, 20, 40, or 80 mg/kg)for 8 weeks (5 days/week)	microglia -mediated regulation of neurogenesis(directly)
(195)		Melatonin	mice	CMS for 7 weeks	I. p (2.5mg/kg) for 4 weeks	microglia -mediated regulation of neurogenesis(directly)
(196)		Omega-3 fatty acids	hippocampal progenitor cell	bilateral oophorectomy	PUFA treatment (approximately 340 mg/g for EPA, 240 mg/g for DHA) for 10 weeks	microglia -mediated regulation of neurogenesis(directly)
(197)		Omega-3 fatty acids	mice	I. p of LPS (1 mg/kg)	feed with n-3 PUFA balanced	microglia -mediated regulation of neurogenesis(directly)

CLP, Cecal ligation and puncture; CMS, Chronic mild stress; CUMS, Chronic unpredictable mild stress; CUS, Chronic unpredictable stress; I. g, intragastric administration; I. p, intraperitoneal injection; MS, Maternal separation; RSD, Repeated social defeat; UCMS, Unpredictable chronic mild stress.

### 5.1 First-line antidepressant drugs in clinical practice

#### 5.1.1 TCAs

TCAs comprise the main category of antidepressant drugs commonly prescribed to treat depression (198). TCAs can inhibit the presynaptic reuptake of 5-HT and norepinephrine, increase the density of monoamine transmitters in the synaptic cleft, and clinically improve depressive symptoms (199). The commonly available TCAs include imipramine, clomipramine, and amitriptyline. Recently, TCAs have been demonstrated to have therapeutic effects of anti-inflammatory and neuroprotection by inhibiting the activation of microglia and promoting neurogenesis in the hippocampus in depression (174, 200). For example, imipramine was found to reduce stress-induced inflammation and depression-like behavior by modulating microglia activation (175). In addition, imipramine inhibits the activation of M1 microglia and the freeing of pro-inflammatory factors to reduce neuronal damage and upregulate BDNF expression to promote neurogenesis (177). However, it is important to note that imipramine is a tricyclic antidepressant known to lower seizure threshold, so caution should be exercised when prescribing it to individuals with epilepsy or those at risk of seizures. Anthor study found that Imipramine has the potential to exacerbate suicidal thoughts during the early stages of administration in patients under the age of 24 (201). Furthermore, Imipramine, along with other types of antidepressants, can potentially trigger the onset of manic or mixed episodes in individuals diagnosed with bipolar disorder (202, 203). Similarly, clomipramine has demonstrated the ability to reduce depressive behavior and neuroinflammation by modulating the microglial NLRP3 inflammasome (176). In another study, Zhang et al. found that clomipramine modulated the microglial NLRP3 inflammasome to reduce depressive behavior and increase hippocampal volume in exposure to CUMS model of rats (204). Meanwhile, amitriptyline was found to inhibit LPS-induced microglial expression of pro-inflammatory factors (205) and increase BDNF expression in microglia (206). It is crucial to recognize that while these TCAs offer potential benefits in treating depression, there are associated risks. For instance, the use of clomipramine during pregnancy has been linked to adverse effects in fetuses, including lethargy and the potential for congenital heart defects, as well as withdrawal symptoms in newborns (207). Additionally, clomipramine may be detected in breast milk, necessitating careful consideration in breastfeeding mothers (207, 208). Amitriptyline, another TCA, has also shown promise by inhibiting microglial expression of pro-inflammatory factors and increasing BDNF expression in microglia (209). However, it’s crucial to note that Amitriptyline should never be combined with alcohol or other central nervous system depressants, as this may significantly potentiate their effects. Additionally, caution must be exercised when transitioning from monoamine oxidase inhibitors to Amitriptyline, as doing so within a two-week timeframe can lead to a potentially life-threatening condition known as serotonin syndrome (41). While TCAs come with various risks and potential side effects, these data suggest that some TCAs hold the potential to not only regulate microglial functions for anti-inflammatory effects but also to promote neurogenesis. As discussed earlier, TCAs can inhibit the presynaptic reuptake of serotonin and norepinephrine, leading to an increased concentration of monoamine transmitters in the synaptic cleft. However, it’s important to note that the precise mechanisms governing microglial activation and neurogenesis promotion remain unclear and warrant further investigation.

#### 5.1.2 SSRIs

Currently, SSRIs are the first line of antidepressants used in clinical practice. This category includes sertraline, paroxetine, fluvoxamine, fluoxetine, citalopram, and escitalopram, which selectively inhibit 5-HT reuptake by antagonistically binding presynaptic membrane 5-HT transporters in neurons, thus increasing the concentration of 5-HT in the synaptic cleft (204, 210). Previous studies have shown that adult neurogenesis and olfactory memory are positively regulated by fluoxetine. It is reported that increased release of pro-inflammatory cytokines by microglia and activation of M1 microglia may lead to elevated expression of serotonin transporter (SERT) (211). Interestingly, SSRIs not only target SERT by inhibiting serotonin reuptake but also centrally act on microglial cells that respond to various signals of inflammatory factors (212). Similarly, fluoxetine cannot improve behavior by inhibiting SERT, which is observed that the increasing the deprivation of dopaminergic neurons and the hurtful activation of microglia in the substantia nigra pars compacta during LPS-induced neurotoxicity (213). These findings support the notion that SSRIs reduce microglial activation by modulating SERT and improving depressive symptoms (214–216). The protective role of SSRIs in neurogenesis has been found to be closely related to the 5H-T and BDNF signaling pathways in schizophrenic models (217). There are no existing studies showing that SSRIs modulate 5-HT to further modulate microglial activation, improve neurogenesis, or alleviate depression. Interestingly, it has been shown that escitalopram before and after treatment can prevent CA1 neuronal death induced by cerebral ischemia by increasing BDNF and thereby reducing microglia activation and oxidative stress (218). Troubat et al. found that the administration of fluoxetine to CUMS mice effectively reduced the activation of microglia in the DG area of the hippocampus of CUMS mice, alleviated neurogenesis damage in the hippocampus, and reduced Depression-like phenotype in CUMS animal model (178). Silvia Alboni et al. obtained the same results (219). SSRIs often recommended as first-line antidepressants, typically take 2 to 4 weeks to initiate therapeutic effects, reaching their maximum efficacy within 4 to 8 weeks (220). However, these benefits come with a range of potential side effects, including gastrointestinal symptoms, hepatotoxicity, weight gain, and metabolic irregularities, as well as the possibility of cardiovascular complications (221). What’s noteworthy is that fewer than 50% of patients do not experience complete relief from their depressive symptoms when treated with initial SSRI therapy (222). This limitation necessitates a transition to second-line treatment options. Furthermore, it’s essential to recognize that the use of antidepressants carries an elevated risk, especially in individuals predisposed to bipolar disorder, of experiencing manic or hypomanic episodes (223). In the future, the promising potential of SSRIs to foster adult hippocampal neurogenesis through their modulation of microglial cells holds great significance. While this avenue shows considerable therapeutic promise, it is essential to address and mitigate the potential side effects associated with SSRIs. As we continue to delve into the intricate mechanisms of neurogenesis and microglial activation, the path forward involves refining the use of SSRIs to maximize their benefits in alleviating depression while minimizing adverse effects. Additionally, ongoing research endeavors aim to uncover novel approaches that harness the neurogenic advantages of SSRIs, ultimately paving the way for more effective and safer antidepressant treatments.

### 5.2 Potential anti-inflammatory candidate drugs

#### 5.2.1 Minocycline

A recent meta-analysis of clinical trials assessed t antidepressant effects and side effects of pharmacological anti-inflammatory interventions in depression or depressive symptoms, revealing that anti-inflammatory agents could enhance the treatment effects of minocycline (224)Minocycline, one of the most studied anti-inflammatory agents, is a second-generation semi-synthetic tetracycline derivative that exhibits efficient spectrum antibacterial effects (70) Further research has found that minocycline exerts its antidepressant effect mainly by inhibiting the activation of microglia, thus protecting hippocampal neurogenesis. For instance, Laumet et al. showed that inflammatory cytokines, including IL-6 and TNF-α, released by hyperproliferative microglia in the hippocampus with peripheral nerve injury could induce secondary changes in hippocampal neurons, thus leading to depression-like phenotype. These effects were reversed by administration of minocycline (225). More importantly, minocycline could alleviate depression-like behaviors induced by early stress in adolescent mice by inhibiting microglial activation and restoring neurogenesis in the hippocampus (100). Additionally, Wadhwa et al. found that minocycline promotes different stages of neurogenesis, such as proliferation (BrdU, Ki-67), differentiation (DCX) cells, and growth factor (BDNF), by inhibiting microglial activation (226). Another study further showed that minocycline decreased M1 microglial marker protein (CD68 and CD16) expression and increased M2 microglial marker protein (CD206 and Arg-1 protein) expression, resulting in a significant increase in neuronal proliferation via the BDNF/TrkB signaling pathway (227). While these findings are promising, it’s important to acknowledge that the current body of evidence is based on a limited number of studies with relatively small sample sizes (70). Therefore, while Minocycline offers a proof-of-concept for treating depression through anti-inflammatory mechanisms, further large-scale clinical trials are necessary to establish its efficacy and safety definitively. These future studies can also explore which subgroups of patients are most likely to benefit from Minocycline-based treatment, possibly based on their microglial markers, inflammatory markers regulating by microglial and neurogenesis markers. In summary, Minocycline presents a compelling candidate for depression treatment due to its anti-inflammatory properties, microglial modulation, and promotion of neurogenesis. Continued research in this direction holds the potential to refine its role in depression therapy and provide tailored solutions for individuals struggling with this debilitating condition.

#### 5.2.2 Ketamine

Ketamine, an N-methyl-d-aspartate receptor antagonist, is considered one of the most promising new agents for antidepression because of its fast and long-lasting antidepressant effects (228, 229). Nonetheless, the antidepressant mechanisms of ketamine remain unclear. Recently, our group found that (R)-ketamine exerts an antidepressant effect by inhibiting microglial ERK-NRBP1-CREB-BDNF signaling in the mPFC of CSDS mice, suggesting that the regulation of ketamine involves microglia and BDNF (230). However, it is unclear whether ketamine affects neurogenesis in the hippocampus. A previous study showed that treatment with ketamine or its metabolite hydroxynorketamine could increase the immune-related protein STAT, after which STAT3 enters the nucleus to regulate downstream transcription, upregulates BDNF, PSD95, and syn1, and alter neuronal plasticity (231, 232). This indicates that ketamine, or its metabolite, plays a crucial function in the modulating of immune-related neuronal plasticity. Notably, another study found that ketamine rapidly relieved depressive-like behaviors by increasing the differentiation of neural progenitor cells and promoting the maturation of new neurons in the DG of the hippocampus (179, 180). Interestingly, another study indicated that ketamine significantly increased the proliferation of NSCs in the DG of model mouse and thus exerted an antidepressant effect but had no effect on synaptic plasticity or hippocampal function (226, 233). While ketamine does offer antidepressant effects, its potential for addiction raises concerns (234). So, the necessity for healthcare supervision in the administration of intravenous ketamine or intranasal esketamine creates a clinical challenge, limiting access for many healthcare providers and their patients. Together, these studies indicate that the antidepressant effect of ketamine may act through a mechanism that modulates microglia and neurogenesis in the hippocampus; however, this mechanism remains to be further studied.

### 5.3 Natural compounds

#### 5.3.1 Xanthoceraside

Xanthoceraside, a triterpenoid saponin monomer, was extracted from the husk of *Xanthoceras sorbifolia Bunge*. Xanthoceraside has been demonstrated to have a broad protective effect against spatial memory impairment, oxidative stress, and inflammatory reactions (235). Guan et al. demonstrated that xanthoceraside could exert antidepressant effects in several depression models of mice by reversing the CUMS-induced inhibition of the hippocampal BDNF signaling pathway and neurogenesis (181), suggesting that the effect of xanthoceraside on depression is related to the protection of neurogenesis. Moreover, xanthoceraside, a triterpenoid saponin monomer compound, has excellent anti-inflammatory effects by regulating the microglia phenotype. It has also been shown that xanthoceraside can inhibit pro-inflammatory cytokine expression in Aβ25-35/IFN-γ-stimulated, and thus has a good effect on the inhibition of microglial activation (182). In summary, while there is currently limited information on specific mechanisms in xanthoceraside in regulating neurogenesis via microglia, existing research suggests that xanthoceraside may be a potential drug candidate for depression because of its actions in promoting neurogenesis and enhancing neuroplasticity, and reducing neuroinflammatory reactions mediated by microglia that contribute to depressive symptoms. However, further research is needed to fully understand its efficacy and underlying mechanisms.

#### 5.3.2 Curcumin

Curcumin is a natural substance extracted from the spice turmeric (*Curcuma longa*), a member of the ginger family (Zingiberaceae). Curcumin exhibits various pharmacological properties, such as antioxidant, regulating microglial phenotype, antineoplastic, hypoglycemic, immunomodulatory, and antimicrobial effects (236, 237). Numerous studies have shown that curcumin administration has an obvious antidepressant effect. The mechanism underlying the antidepressant activity of curcumin may involve the regulation of the serotonin and dopamine system, anti-inflammatory effects, and neuroprotection (219, 238). Previous studies have speculated that neurogenesis and neuroprotection in susceptible brain areas may have a critical function in psychiatric and neurological disorders, including anxiety depression, and Alzheimer’s disease (239). Recently, it has been shown that treatment with curcumin could reverse hippocampal neuron damage in response to chronic stress and increase cell proliferation and NSCs populations, suggesting that curcumin relieves impaired hippocampal neurogenesis (240). Curcumin also exerts a regulatory effect on microglial phenotypes. For example, curcumin has been shown to significantly alleviate LPS-induced inflammation by switching from the M1 pro-inflammatory phenotype to the M2 anti-inflammatory phenotype by downregulating the TLR4/NF-κB pathway (183, 241). Current research suggests that curcumin has the potential in improving depression by regulating microglia. Moreover, it also promotes neurogenesis, which further alleviates depressive symptoms. However, there is currently no direct evidence to prove that curcumin promotes neurogenesis by modulating microglia. Therefore, more in-depth research is needed to explore the mechanism of curcumin in regulating microglia and promoting neurogenesis, and to further demonstrate it as a drug candidate for treating depression.

#### 5.3.3 Paeoniflorin

Paeoniflorin is a water-soluble monoterpenoid glycoside derived from multiple herbaceous plants such as *Radix Paeoniae Rubra*, *Radix Paeoniae Alba*, *Paeonia suffruticosa*, and *Cimicifugae Foetidae*. Many studies have demonstrated that paeoniflorin possesses multiple pharmacological properties including improving microglial-mediated inflammatory factors, antidepressant, neuroprotective, and anti-apoptotic effects (184, 242). A systematic review and meta-analysis revealed that paeoniflorin can significantly improve depressive-like behaviors in animals, suggesting that paeoniflorin can be a potential treatment for patients with depression (243). First, the antidepressant effects of paeoniflorin may be related to its neuroprotective effects. For example, Sicheng et al. found that paeoniflorin reversed the loss of dendritic spine density and the decline of the expression of BDNF and PSD95 in the hippocampus of CUMS model mouse, thus exerting an antidepressant effect (185). More importantly, paeoniflorin inhibited microglial proliferation by suppressing the JNK and NF-κB signaling pathways, leading to significant reductions in the pro-inflammatory cytokines IL-1β, IL-6, and TNF-α mediated by microglia (186). Compared to the control group, paeoniflorin promoted the expression of von Willebrand factor and doublecortin, suggesting that Paeonia extract contributes to neurogenesis and angiogenesis in rats (186). Paeoniflorin has excellent anti-inflammatory effects. The data indicate that paeoniflorin exerts antidepressant effects and prevents neuroinflammation by inhibiting the CASP-11-mediated pyroptosis signaling pathway activated in overactivated small glial cells in the hippocampus of reserpine-treated mice, resulting in synaptic plasticity abnormalities (184). *In vitro* experiments with N9 microglial cells of mice show that paeoniflorin can also prevent LPS and adenosine triphosphate (ATP)-induced pyroptosis and has an effective and selective CASP-1 activator inhibitor VX-765 to promote the inhibitory effect of paeoniflorin on pyroptosis (184). This reveals a previously unrecognized inflammatory mechanism of antidepressant action, and proposes a unique treatment opportunity to relieve depression through paeoniflorin therapy. Interestingly, there is direct evidence that paeoniflorin can protect neurogenesis by regulating the activation of microglia, modulating M1/M2 subset polarization in the hippocampus, and inhibiting the freeing of inflammatory cytokines (187). These results indicated that the regulation of microglia and hippocampal neurogenesis is an important mechanism underlying the antidepressant effects of paeoniflorin.

#### 5.3.4 Resveratrol

Resveratrol is a polyphenol and phytoalexin derived from the skin of grapes, red wine, Japanese knotweeds, and peanuts. It has been shown that resveratrol has extensive properties including antioxidant, improving microglial-mediated inflammatory factor, and neuroprotective properties (244–246). Resveratrol showed excellent anti-depressive activity with an obvious improvement in depressive-like behavior tests. Alyssa et al. identified three main biological mechanisms of resveratrol, including modulating the HPA axis, microglia, and BDNF and neurogenesis, based on results from 22 preclinical studies (244). In terms of anti-inflammatory properties, resveratrol can inhibit the M1 microglial activation that induced by LPS stimulation, which involves the balance of M1/M2 polarization and the NLRP3 inflammasome (188–190). In terms of neurogenesis, resveratrol could exhibit neuroprotective and hippocampal neurogenesis properties (247). For example, pretreatment with resveratrol was found to activate the Sirt1 pathway, inhibit microglial activation, promote neurogenesis indicated by BrdU^+^DCX^+^ markers, and improve depressive-like behaviors in LPS-treated mice (191). Similarly, Wu et al. found that functional fermented milk rich in resveratrol significantly enhanced neurogenesis in a D-galactose mouse model (192). It can be concluded that resveratrol could prevent mood dysfunction by increasing hippocampal neurogenesis and reducing glial activation, but the detailed mechanisms remain to be elucidated.

While short-term animal experiments have provided strong support for the potential of natural compounds such as xanthoceraside, curcumin, paeoniflorin, and resveratrol in promoting adult hippocampal neurogenesis mediated by microglial cells as a strategy against depression, there is currently a lack of additional data revealing the potential toxic side effects of these drugs. To gain a more comprehensive understanding of the potential value of these compounds, further animal experiments are needed. Furthermore, these studies should place particular emphasis on investigating the effects of repeated long-term administration of these compounds, including in-depth research into organ toxicity, immune responses, and tolerance. This collective effort aims to ensure the success and safety of these natural compounds in the treatment of depression, providing a more reliable foundation for future clinical practices.

### 5.4 Others

#### 5.4.1 Glycogen synthase kinase 3 inhibitor

Glycogen synthase kinase 3 (GSK-3) is a serine/threonine kinase enzyme, which has two subunits, including GSK-3α and GSK-3β, the dysregulation of which could lead to mental disorders, including depression and cognitive impairment (248, 249). It has been showed that a significant increase in the activation of GSK-3β has been found in postmortem brain regions of depression patients (249). As a critical regulator of cognitive function, GSK-3β plays a significant role in cognitive processes, including synaptic plasticity, neurogenesis, and neural cell survival (250). *In vitro* and *in vivo* studies have shown that GSK-3 inhibits neurogenesis in the adult hippocampus. In contrast, adult hippocampal neurogenesis is promoted by the inhibition of GSK-3 (251). These results demonstrate that the development of a GSK-3 inhibitor will be beneficial for adult hippocampal neurogenesis. Furthermore, it has been found that the mechanism by which GSK-3 regulates cognitive functions involving neurogenesis, synaptic plasticity, and neural cell survival is related to neuroinflammation. Microglia-mediated inflammatory responses are highly dependent on GSK-3 activity. Studies have shown that GSK-3 has pro-inflammatory effects (252), whereas GSK-3 inhibitors exhibit anti-inflammatory effects by upregulating the release of IL-10 from microglia (253). Therefore, based on the effect of GSK-3 on neurogenesis and microglia, we propose GSK-3 inhibitor as a potential therapeutic agent for depression.

#### 5.4.2 Melatonin

Melatonin, called N-Acetyl-5-methoxytryptamine, is a hormone primarily synthesized and secreted by the pineal gland, and has various biological activities, which contain the modulating of the biological clock, anti-inflammatory, analgesic, anti-depressive, and neuroprotective effects (254). Melatonin exhibits antidepressant effects in multiple animal models, but its mechanism remains unclear (255). Inhibition of neuroinflammation and promotion of neuroplasticity may be the most important mechanisms by which melatonin maintains antidepressant-like effects (256). Melatonin can improve LPS-induced acute depression-like phenotype by suppressing the activation of the microglial NLRP3 inflammasome, thus reducing the pro-inflammatory factors released by microglia (193). In another study, melatonin was found to not only inhibit the M1 microglial activation as well as the NLRP3 inflammasome, but also unregulate the BDNF-ERK-CREB pathway to promote neurogenesis and reduce synaptic loss in the hippocampus (194). Similarly, in CMS rats, melatonin has been found to inhibit inflammatory cytokine production and protect neurogenesis, thus ameliorating depressive symptoms (195). These results suggest that the antidepressant mechanism of melatonin may be engaged in microglial activation and neurogenesis. Although the correlation between melatonin and depression is complex, exploring the effects of melatonin on microglia and neurogenesis in depression may facilitate the development of new antidepressant candidates.

#### 5.4.3 Omega-3 fatty acids

Omega-3 fatty acids are derived from α-linolenic acid, which is mainly obtained from the diet because of its failure to be synthesized by humans. Omega-3 fatty acids, including eicosapentaenoic acid (EPA) and docosahexaenoic acid (DHA), are incorporated into cell membranes and play a significant role in anti-inflammatory processes and the viscosity of cell membranes (257). A meta-analysis verified that low levels of omega-3 fatty acids are strongly associated with the development of mood disorders, while omega-3 fatty acid ingest is related to alleviated depressive symptoms (258). The ovariectomy procedure induces anxiety and depression-like behaviors, accompanied by increased neurodegeneration in the hippocampal region and activation of microglia in rats (196). Additionally, ovariectomy enhances the expression of pro-inflammatory cytokines and suppresses the expression of anti-inflammatory cytokine IL-10 (196). Correspondingly, ovariectomy strengthens the NFκB signaling pathway and shifts microglia polarization from the anti-inflammatory M2 to the pro-inflammatory M1 phenotype (196). However, daily supplementation with omega-3 polyunsaturated fatty acids can inhibit the M1 polarization of microglia in ovariectomy rats and increase the M2 polarization (196). Moreover, omega-3 fatty acids also show a preventive effect on glucocorticoid-induced reduction in hippocampal neurogenesis and increase apoptosis (259). Maternal intake of omega-3 fatty acids can affect hippocampal neurogenesis during development, and this effect may persist into adulthood through alterations in adult hippocampal neurogenesis. Injection of bacterial endotoxin LPS in mice reduced the number of neural progenitor cells, and this effect was exacerbated by a diet deficient in omega-3 fatty acids. A diet deficient in omega-3 fatty acids also reduced DG volume, decreased adult hippocampal neurogenesis (BrdU^+^ DCX^+^ positive cells), and decreased the number of microglia Administration of omega-3 fatty acids reversed these effects (197). These findings suggest that a diet deficient in omega-3 fatty acids has negative effects on the cellular composition of the adult DG, reduces adult hippocampal neurogenesis, and impairs the monitoring of microglia. It is reported that omega-3 fatty acids significantly reduce the excretion of pro-inflammatory cytokines, including IL-6, IL-1β, and TNF-α, via microglia and have an enhanced anti-inflammatory effect (260). The influence of omega-3 fatty acids on microglia may be a mechanism underlying its antidepressant effects. Therefore, according to the multiple effects on hippocampal neurogenesis and anti-inflammatory effects on microglia, omega-3 fatty acids will be a valuable and safe dietary product in the development of antidepressant agents.

Considering the research on GSK-3 inhibitors, melatonin, and Ω-3 fatty acids in animal models of depression, these therapeutic approaches presently encounter various mysteries and knowledge gaps within the field of depression. Although some potential therapeutic mechanisms have been demonstrated at the cellular and molecular levels, further exploration is essential to fully uncover their potential and the associated risks within the context of the whole organism.

Currently, there are relatively few antidepressant mechanisms and drug candidates that focus on regulating adult hippocampal neurogenesis through the modulation of microglia. The aforementioned pre-clinical and clinical studies provide important ideas and insights for the treatment of depression, particularly in cases related to impaired neurogenesis. Further investigation of specific targets that regulate hippocampal microglia may offer more effective options for drug therapy. These research findings reveal the important role of microglia in hippocampal neurogenesis, providing new avenues for the development of novel drug candidate treatments for depression. Future research should focus on optimizing these drugs and determining their mechanisms of action, in order to more effectively translate these therapeutic methods into clinical practice.

## 6 Conclusions and perspectives

Depression is a common psychiatric disorder with high morbidity and suicidality that adversely affects an individual’s life and is closely related to a decreased life span and impaired quality of life (121). Although decades of research have largely focused on serotonergic dysfunctions in the synaptic cleft, first-line antidepressants designed for the target have not yet received satisfactory results due to the deficiency in patients’ responses and serious side effects (261). The main reason for this might be the incomplete understanding of the pathogenesis of this disease.

Adult hippocampal neurogenesis is capable of producing new functional neurons to form supplementary neural connections with other regions of the hippocampus, supporting spatial cognitive function and emotion under physiological conditions. Altered adult hippocampal neurogenesis plays an important role in the development and treatment of depression (262). Microglia, the predominant resident immune cells in the brain, constitute the brain microenvironment that mainly regulates adult hippocampal neurogenesis. The important role of microglia in adult hippocampal neurogenesis under physiological conditions has been demonstrated in several studies. For instance, microglia maintain the neurogenic niche environment through their phagocytic capacity and interaction with neurons through fractalkine-CX3CR1 signaling (263). In addition, microglia secrete growth factors and cytokines to support the development of new neurons in the hippocampus (264). However, the exact molecular mechanisms by which microglia regulate adult hippocampal neurogenesis and contribute to depression remain to be elucidated.

In this review, we illustrate the roles of microglia and adult hippocampal neurogenesis in depression, as well as the possible mechanism of crosstalk between the two. Finally, we summarize potential therapeutic approaches that focus on this mechanism. To draw a conclusion upon the review, when depression occurs, the function of microglial activation in adult hippocampal neurogenesis is similar to a double-edged sword, exerting both protective and detrimental effects. This depends on how the pathological environment determines the fate of microglial phenotypes. In line with this, promising therapies have reversed the shift in microglial phenotypes to those that contribute to the improvement of depression. In clinical trials, heterogeneity among patients with depression is common due to factors such as age, gender, medical history, and severity of symptoms (265). For example, in approximately 52% of untreated patients with depression, a single intervention such as antidepressant medication or evidence-based psychological therapy is insufficient for full remission, with most patients only experiencing partial symptom relief due to patient heterogeneity (266). Among patients participating in “real-world” clinical trials, the combination of patient heterogeneity was 28% (267). In addition, patients with increased inflammatory markers are more likely to be treatment-resistant to commonly used antidepressants. Patients with autoimmune disorders have a high prevalence of depression, and are at increased risk for subsequent autoimmune disorders such as rheumatoid arthritis, multiple sclerosis, inflammatory bowel disease, and systemic lupus erythematosus (268–270). Therefore, this is why about 50% of patients with depression respond to first-line treatment with SSRIs (271). Here, although other candidate drugs can improve microglia function to combat depression, we believe that minocycline, ketamine, and natural compounds paeoniflorin have the most potential, as they can directly regulate microglia function across the blood-brain barrier to enhance neurogenesis and exert antidepressant effects. We expect that our research will have positive implications for the development of future drugs for depression treatment. Therefore, newer genetic, epigenetic, and high-throughput omics technologies are expected to be applied to explore the molecular, cellular, and circuits involved in depression, and to discover novel therapeutic strategies aimed at promoting mechanisms in the future.

## Author contributions

SYF, ZBW wrote the manuscript, and they contributed equally to this work. YLG, WJZ, CMW, NJY, JBC, WZH, XWM and XFG helped to collect and modify this manuscript. JXC and XJL conceived, designed and revised the manuscript. LLF prepared figures and tables, and revised the manuscript. All authors read and approved the final manuscript.

## Glossary

**Table:**

HPA axis	Hypothalamic-pituitary-adrenal axis
CSF	Cerebrospinal fluid
CNS	Central nervous system
NLRP3	Nod-like receptor protein 3
NLR	Nucleotide-binding oligomerization
IL-1β	Interleukin-1β
IL-6	Interleukin-6
TGF-β	Transforming growth factor-β
IL-10	Interleukin-10
IL-4	Interleukin-4
IL-13	Interleukin-13
IFN-α	Interferon-α
IFN-γ	Interferon-γ
CD163	Cluster of differentiation 163
CD206	Cluster of differentiation 206
M1 microglia	M1-phenotype microglia
INOS	Inducible nitric oxide synthase
CD86	Cluster of differentiation 86
MS	maternal separation
CUMS	Chronic unpredictable mild stress
CMS	Chronic mild stress
CSDS	chronic social defeat stress
CUS	Chronic mild stress
CRS	Chronic restraint stress
LPS	Lipopolysaccharide
TLR4	Toll-like receptor 4
AD	Alzheimer’s disease
PFC	Prefrontal cortex
DG	Dentate gyrus
NSC	Neural stem cell
SVZ	Subventricular zone
SGZ	Subgranular zone
5-HT	5-hydroxytryptamine
NF-κB	Nuclear transcription factor-kappa B
MyD88	Myeloid differentiation factor 88
PRRs	Pattern recognition receptors
NLRs	Nucleotide-binding oligomerization domains leucine-rich repeat-containing protein receptors
R-SDS	Repeated social defeat stress
TLR2/4	Toll-like receptor 2 and 4
TNF-α	Tumor necrosis factor α
BDNF	Brain derived neurotrophic Factor
ERF	Extracellular signal-regulated kinase
CREB	cAMP-response element binding protein
MIP2	Macrophage inflammatory protein-2
CX3CR1	C-X3-C motif chemokine receptor 1
BrdU	5-ethynyl-2-deoxyuridine
TRY	Tryptophan
SSRIs	Selective serotonin reuptake inhibitors
DCX	Doublecortin
IDO	Indoleamine 2,3-dioxygenase
TDO	Tryptophan 2,3-dioxygenase
JAK	Janus kinase
STAT	Signal transducer and activator of transcription
KYN	Kynurenine; TrkB: Tyrosine kinase receptor B
KLF4	Kruppel like factor 4
CDKL5	Cyclin dependent kinase like 5
Brdu	5-Bromo-2-deoxyuridine
CAMP	Cyclic adenosine monophosphate
Tnfr1	Tumor necrosis factor receptor 1
GR	Glucocorticoid receptor
PD	Parkinson disease
QUIN	Quinolinic acid
miRNA	MicroRNA
ncRNA	non-coding RNA
KLF4	Kruppel like factor 4
HNK	Hydroxynorketamine
ALA	α-linolenic acid
EPA	Eicosapentaenoic acid
DHA	Docosahexaenoic acid
Aβ25–35	Amyloid b-Protein Fragment 25-35
GSK-3	Glycogen synthase kinase 3
NO	Nitric oxide
HD	Huntington’s disease
GCS	Glucocorticoid
DST	DEX inhibition test
ACTH	Adrenocorticotropic hormone
SGK	Serum and glucocorticoid induced kinase
NSCs	Neural stem cells
Arg-1	Arginase-1
GF	Germ-free
IGF-1	Insulin-like growth factor-1
CCL17	Chemoattractant cytokine ligand 17
CCL18	Chemoattractant cytokine ligand 18
PSD95	Postsynaptic density protein 95
ERK	Extracellular signal-regulated kinase
TCAs	Tricyclic antidepressants
SERT	serotonin transporter
